# The 10 top prescribed medicines in Germany from 1985 to 2022: pharmacological analysis

**DOI:** 10.1007/s00210-024-03615-5

**Published:** 2024-11-21

**Authors:** Lennart Schröder, Roland Seifert

**Affiliations:** Institute of Pharmacology, Hannover Medical School, D-30625 Hannover, Germany

**Keywords:** Pharmaceutical expenditure, Healthcare system, Statutory health insurance

## Abstract

For many years, pharmaceutical expenditure has been the second largest cost item for statutory health insurance funds (SHI) in Germany after hospital costs. Since prescriptions and expenditure on medicines play such a major role in the German healthcare system, the question arises as to what causes changes in prescriptions. To answer this question, the prescribing trends for the top 10 drugs in 2022 were analyzed over a period of 38 years, from 1985 to 2022. The prescribed defined daily doses (DDD) and the costs per defined daily dose for the 10 medicines were taken from the Arzneiverordnungsreport (AVR) from 1986 to 2023, and the changes in prescribing behavior and their causes were analyzed. The ten most important medicines in 2022, accounting for over 41% of all prescribed daily doses, were ramipril, candesartan, pantoprazole, amlodipine, atorvastatin, levothyroxine, torasemide, simvastatin, bisoprolol, and metoprolol. There are many different reasons for an increase in prescriptions, such as the introduction of generics, a positive study, or a price reduction. Further reasons for an increase in prescriptions are an extension of the indication or the recall of a competing medicine. A change in guidelines or the increasing treatment of laboratory values without clinical symptoms can also lead to an increase in prescriptions. There are also many different reasons for a drop in prescriptions, such as the generic launch of a competitor medicine or a positive study for a competitor medicine. Other reasons for a drop in prescriptions are a negative study or a discussion about the use of a drug. Sometimes, the reasons for prescription changes are also irrational. Overall, this is the most comprehensive long-term analysis of drug prescriptions in Germany. Our data is helpful for predicting drug prescriptions and for preventing future drug shortages not only in Germany but also worldwide.

## Introduction

Costs for drug prescriptions are a major factor in healthcare systems worldwide. The causes of changes in the prescription of a drug are largely known, such as the fact that the introduction of a generic drug (Blankart and Vandoros 2024) or a positive evaluation by the Federal Joint Committee (GBA) (Blankart and Stargardt 2020) usually leads to an increase in prescribing. It is also known how the recall of a drug affects competing drugs (Rudolph et al 2021). However, it is currently unclear whether there are other causes of prescription changes. Furthermore, due to the limited study duration, the studies do not analyze how the events that lead to prescription changes have affected prescriptions in the long term.

A US study analyzed national trends in medication use in practice from 1985 to 1999 (Burt 2002), but did not focus on the causes of the increase. Other studies, such as an analysis of opioid prescriptions over 15 years (Olfson et al. 2013), an analysis of the “Trends in Systemic Glucocorticoid Utilization in the United Kingdom from 1990 to 2019” (Menzies-Gow et al. 2024), or an analysis of the “Treatment pattern trends of medications for type 2 diabetes in British Columbia, Canada” (Carney et al. 2022), also only looked at one drug group or indication. In addition, most studies only examined a relatively small study population.

In Germany, there has not yet been an analysis that examines drug prescriptions over a longer period, although drug expenditure has been the second largest cost block of statutory health insurance funds (SHI) in Germany after hospital costs for many years (Ludwig et al. 2023). Although the Arzneiverordnungsreport (AVR) discusses the current prescriptions of drugs and in some cases also presents the global prescription histories over 10 years for some drugs, these never go back as far as 1985. This analysis therefore examines the question of how various events affect the prescribing behavior of physicians. To determine this, we examined how prescriptions of the 10 most important medicines in 2022 have developed since 1985. This analysis covers a period of 38 years and the prescriptions of all persons with statutory health insurance (73.7 million insured persons in 2022), so this is also an approach to identify potential causes of prescription changes and to show the long-term effects of events on drug prescriptions.

## Material and methods

### Top 10 medicines 2022

**Fig. 1:**
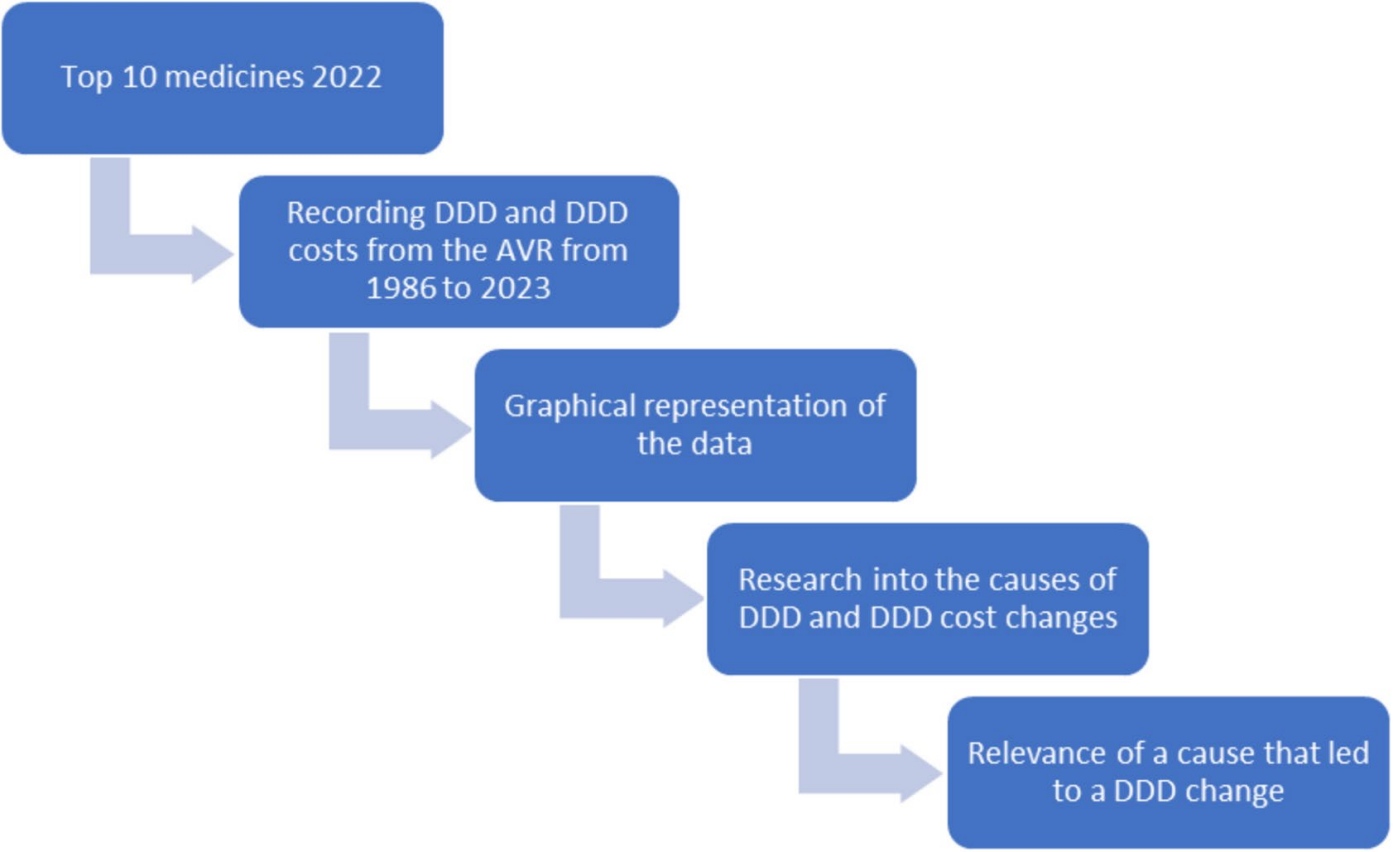
Flowchart of the analytical procedure

Figure 1 shows the methodological approach of our study. For the analysis of the medicine prescription history over 38 years, the top 10 prescribed medicines from the year 2022 were selected. These were provided by WIdO. The 10 most important medicines account for almost 42% of all defined daily doses prescribed in 2022 and have a relatively broad range of indications, making them suitable as representative medicines for an analysis.

### Recording DDD and DDD costs from the AVR from 1986 to 2023

The drug prescription reports were used to collect the data (Fig. 1). The Drug Prescription Report has been published annually since 1985 by authors from pharmacology, clinical practice, health economics, and health insurance and is based on the prescription data of drugs for outpatients of the statutory health insurance (SHI) (Ludwig et al. 2023). Whereas, until 2002, only a random sample was analyzed, since then, all prescriptions from all patients with statutory health insurance in Germany have been analyzed. The Drug Prescription Report analyzes the 3000 most frequently prescribed drugs in Germany, describes the prescriptions of the previous year, and often also names possible causes for changes. The prescribed DDD and the net DDD costs were extracted and tabulated from this report (Schwabe und Paffrath 1986, 1987, 1988, 1989, 1990, 1991, 1992, 1993, 1994, 1995, 1996, 1997, 1998, 1999, 2000, 2001, 2002, 2003, 2004, 2005, 2006, 2007, 2008, 2009, 2010, 2011, 2012, 2013, 2014, 2015, 2016; Schwabe et al. 2017, 2018, 2019; Schwabe and Ludwig 2020; Ludwig et al. 2021, 2022, 2023). In order to guarantee the correctness of this data, it was then randomly checked by a second person.

The decision was made in favor of the prescribed DDD, as a change in pack size or dosage strength cannot falsify the consumption measured in the DDD. Although the DDD for some medicines has also changed over the last 38 years, the DDDs were developed to track the prescribing patterns of medicines, and a change in the DDD has always been clearly visible. In addition, an international comparison with the DDD of the World Health Organization (WHO) has been possible since 1997 at the latest. The defined daily dose is based on the amount of an active substance or drug typically used per day for the main indication in adults (Nordic Council on Medicines 1985) (Schwabe and Paffrath 2000). The DDD cost is the net cost per defined daily dose. If a medicine is available in several variants, for example, after the introduction of generics, the average weighted DDD costs are calculated. These depend on the number of DDDs prescribed, i.e., the more frequently a cost-effective medicine is prescribed, the lower the average net DDD cost of the medicine. The average weighted DDD costs have been abbreviated to DDD costs for better readability.

To make the DDD more comparable, all DDDs were converted to the current DDD of 2022. This was also done with the DDD costs. However, this conversion was not possible for all medicinal products, so this was marked accordingly. In order to make the DDD costs of the medicines even more comparable, the DDD costs before 2001 were converted into euros using the official exchange rate (Bundesbank 2024). Inflation was taken into account. It should be noted that the DDDs in the Drug Prescription Report are only the DDDs prescribed by doctors. However, as all the top 10 medicines are prescription-only, every dose of the medicines has been included in the AVR.

Until 1992, only the DDD from West Germany were analyzed; from 1990 to 1992, prescriptions from the new federal states had their own chapter in the Drug Prescription Report. However, since not all the medicines analyzed were listed in this chapter of the Drug Prescription Report, the DDDs of the new federal states were only included from 1993 onwards. This was then marked accordingly for the medicines that were already on the market before 1993.

### Analyses and presentation of the data

The prescriptions were then displayed graphically using the prescribed DDD in order to better identify changes in doctors’ prescribing behavior. DDD costs were also presented graphically to recognize changes in the cost of a drug. These graphs were then compared with each other to illustrate the impact of a change in DDD costs on the DDDs prescribed. For reasons of space, only the comparative graphs relating to DDD and DDD costs of the top 3 most frequently prescribed medicines were presented in this paper. In addition, the prescription changes of drugs with the same indication or analog preparations were compared with each other in order to investigate whether an event of one medicine, such as an extension of the indication, has an impact on the prescriptions of the other medicine with the same indication and what effect it has. This makes it possible to investigate the reasons for the preference for one drug over another and also to explain changes in prescribing that were often initially unclear.

For the graphical representation of the data, the medicines were divided into their active ingredient groups. However, it should be noted that only the top 10 most frequently prescribed active ingredients are shown here, which means that the many other medicines in the active ingredient group are not shown in the graph.

### Research into the causes of DDD and DDD cost changes

Research into the causes of a change in prescribing behavior was carried out not only with the help of the drug prescription reports but also with the help of other sources. If there was a change in the prescription, the AVRs and the Internet were searched for the cause of this change. In addition, the AVR was searched for possible events that could have an influence on prescriptions, and it was analyzed whether this had led to a change in prescriptions. The same analysis was also carried out for DDD costs.

### Relevance of a cause that led to a DDD change

Finally, a table was used to show whether a particular event always led to a change in prescribing and the extent of this change in prescribing in order to better illustrate the relevance of an event. For this purpose, the percentage change in prescriptions 2 years before the event and the percentage change in prescriptions in the year of the event and the following year were calculated. The following formula was used for this calculation:

$$\left(\frac{A-B}{B}\times 100\right)-\left(\frac{B-C}{C}\times 100\right)$$

A = DDD 1 year after event, B = DDD 1 year before event, and C = DDD 3 years before event.

However, it must also be noted here that the percentage changes in prescriptions can still be influenced by other events and should therefore only be considered in the context of prescription trends.

## Results and discussion

Table 1 provides an overview of the 10 most important drugs in 2022, showing the rank, the year of introduction, the number of DDDs prescribed in 2022, and the share of all DDDs prescribed in 2022. 47,592.31 million DDDs were prescribed on the German SHI market in 2022 (Ludwig et al. 2023). At 19,522.1 million DDD, the top 10 medicines account for 41.9% of total prescriptions in Germany in 2022. All top 10 medicines have more than 850 million prescribed DDD in 2022, and the top 5 all have more than 1.5 billion prescribed DDD.

**Table 1:** Rank, year of introduction, prescribed DDD 2022, and percentage share of all prescribed DDD 2022 of the 10 most important medicines of 2022: created with data from the AVR 2023 and a table of the top 10 medicines of the WIdO

TOP 10	Medicines	Year of introduction	DDD in MIO	Percentage share of all DDD 2022
1	Ramipril	1990	4794.7	10.70%
2	Candesartan	1997	3064.9	6.44%
3	Pantoprazole	1994	2940.1	6.18%
4	Amlodipine	1993	1890.2	3.97%
5	Atorvastatin	1997	1782.3	3.74%
6	Levothyroxine	1926	1458.9	3.07%
7	Torasemide	1992	1023.2	2.15%
8	Simvastatin	1990	912.3	1.92%
9	Bisoprolol	1986	853.5	1.79%
10	Metoprolol	1976	802	1.69%

### ACE inhibitors and AT1R antagonists

**Fig. 2:**
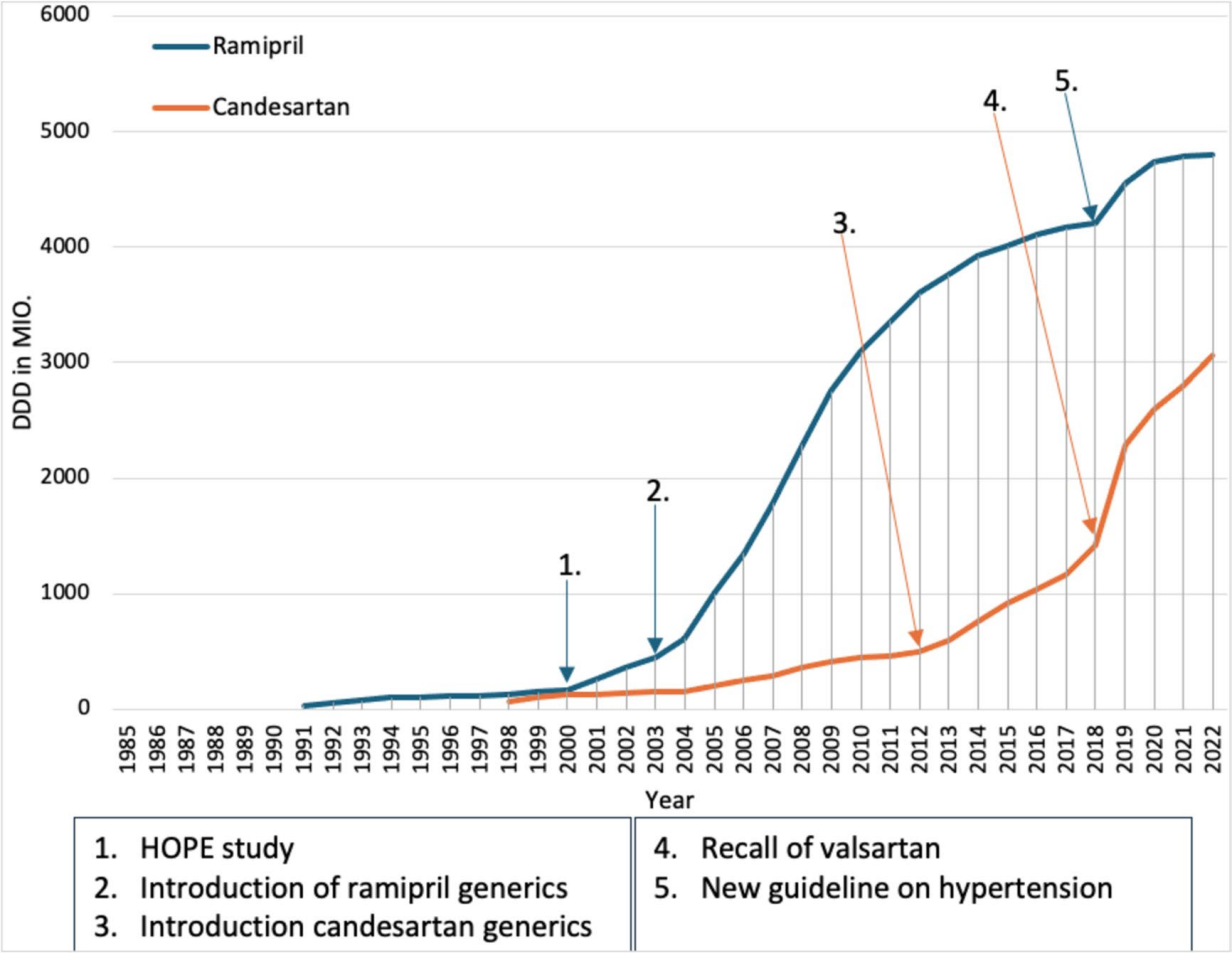
Prescribed DDD of ACE inhibitors and AT1R antagonists from 1985 to 2022: taken from the drug prescription reports from 1991 to 2023—ramipril (blue) and candesartan (orange). Blue arrows refer to a change in the prescription of ramipril. Orange arrows refer to a change in the prescription of candesartan

Ramipril was launched in Germany in October 1990 as a further angiotensin-converting enzyme (ACE) inhibitor alongside captopril, enalapril, and lisinopril and had already reached 19.2 million DDD by 1991 (Fig. 2). This was probably partly because all ACE inhibitors had an increase in prescriptions due to the reduction in morbidity and mortality in heart failure shown in studies (e.g., CONSENSUS Trial Study Group 1987, which showed a reduction in mortality in severe heart failure compared to placebo) and partly because ramipril was the cheapest ACE inhibitor on the German market in 1991. Prescriptions rose steadily in the following years despite pressure to cut the drug budget, showing that doctors may prescribe more expensive medicines if this is necessary for good therapy. In addition, the prescriptions of the former German Democratic Republic (GDR) were considered from 1993 onwards.

From 1996 onwards, prescriptions for ramipril did not increase as much as in previous years. This was probably partly due to the introduction of the inexpensive captopril generics and partly due to the introduction of candesartan in December 1997. Candesartan saw a sharp increase in prescriptions due to its significantly lower DDD cost compared to losartan and valsartan and was already the most frequently prescribed angiotensin II receptor type 1 (AT1R) antagonist in 1998. In addition, the increase in prescriptions is probably due to the lower side effects of AT1R antagonists compared to ACE inhibitors, such as coughing, with similar efficacy (Pitt et al. 2000). This clearly shows that the introduction of a generic analog or the introduction of a new medicine used in the same indication can lead to a reduced increase in prescriptions. However, new medicines sometimes only have a strong increase in prescriptions because some doctors are of the opinion that new medicines are always better than older ones and should therefore be preferred (Schwabe and Paffrath 2000).

In 2000, ramipril again received a strong increase in DDD due to the HOPE study. In the HOPE study, patients at high cardiovascular risk without pre-existing heart failure were treated with ramipril or a placebo. The high-risk patients treated with ramipril had a risk reduction for stroke, myocardial infarction, cardiovascular death, and for all-cause mortality, as well as a reduction in the risk of several other endpoints including heart failure and revascularization procedures (Sleight 2000). This study then led to ramipril being given an extended indication for patients with increased cardiovascular risk in 2002. Here, too, a positive clinical study has presumably led to an increase in prescriptions. The HOPE study and the lower DDD costs of ramipril then probably led to only a slight increase in prescriptions of candesartan after the strong increase in prescriptions in previous years.

In November 2003, the first generic versions of ramipril were also introduced, making ramipril once again the cheapest ACE inhibitor. This most likely led to the sharp increase in the prescription of ramipril. Prescriptions of ramipril continued to rise sharply in the following years, while the DDD of candesartan increased only slowly. The fact that prescriptions of candesartan rose again in 2005 is probably due to the reference price of the SHI Modernization Act, which led to a reduction in DDD costs. The SHI Modernization Act 2004 was intended to reduce SHI expenditure by, among other things, excluding non-prescription medicines from SHI coverage, increasing the co-payment for medicines to 10% of the sales price and setting reference prices for patent-protected medicines without additional benefits, but these did not come into force until 2005 (Schwabe and Paffrath 2005).

In 2012, candesartan generics were then introduced, which most likely led to the increase in prescriptions for candesartan. However, prescriptions of ramipril also continued to rise, albeit not as strongly as in previous years. The generic launches of ramipril and candesartan are good examples of the impact of generic launches on prescriptions. The introduction of own generics led to a sharp increase in prescriptions, while prescriptions for medicines used in the same indication often stagnate or even fall.

The increase in prescriptions for both medicines continued until 2018, with the increase in prescriptions for candesartan increasing more strongly, while that for ramipril leveled off. In 2019, ramipril and candesartan experienced a sudden increase in the number of DDDs prescribed. This was more pronounced for candesartan than for ramipril and is probably due to the recall of valsartan due to impurities (BfArM 2018). However, the new, amended hypertension guideline from 2018 presumably also led to the increase in prescriptions for ramipril and candesartan. While the 2013 hypertension guideline still recommended treating patients with high-normal blood pressure (130–139/85–89 mmHg) and cardiovascular risk factors with lifestyle changes only (Mancia et al. 2013), the updated 2018 hypertension guideline recommended considering drug treatment for these patients in addition to lifestyle changes (Williams et al. 2018). In addition, the 2018 guideline recommends that “blood pressure levels of 130/80 mmHg or lower should be aimed for in most treated patients” (Williams et al. 2018), whereas in the 2013 guideline, only blood pressure levels below 140/90 should be aimed for (Mancia et al. 2013). Presumably, the new guideline has led to more patients being treated with antihypertensives on the one hand and patients who are already being treated receiving higher doses on the other, which shows that a change in the guideline can lead to an increase in prescriptions.

While the increase in ramipril prescriptions leveled off again in 2020, candesartan prescriptions continued to rise. This is presumably due to the lower adverse medicine reactions and thus greater adherence with the same effectiveness (Messerli et al. 2018). Nevertheless, angiotensin receptor antagonists should only be used in cases of intolerance to ACE inhibitors (Williams et al. 2018), as the DDD costs of candesartan are still twice as high as those of ramipril in 2022. However, “the real costs of ACE inhibitors are higher, as ramipril in particular is most frequently prescribed at a higher daily dose (5 mg) than the WHO DDD (2.5 mg)” (Ludwig et al. 2022).

### Proton pump inhibitors

**Fig. 3:**
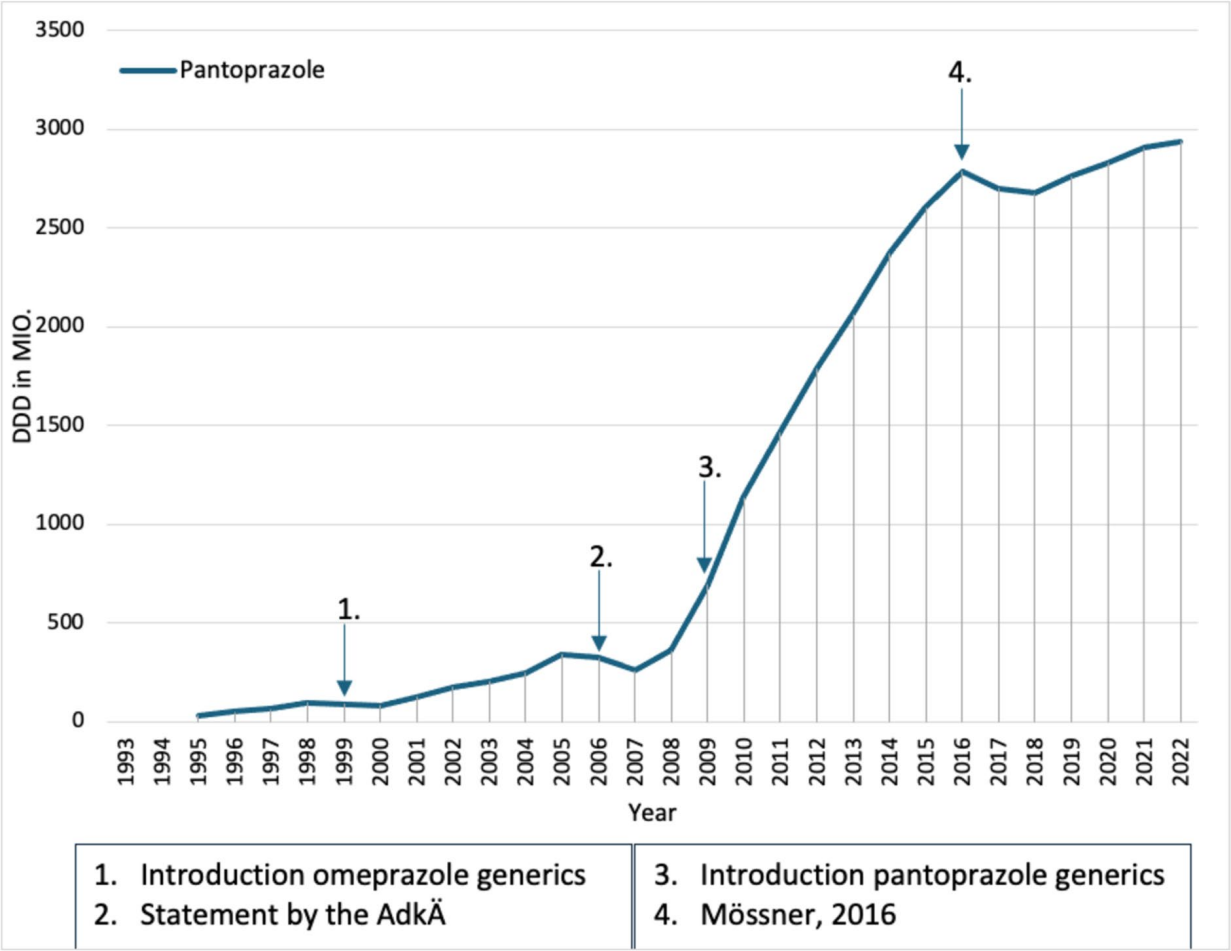
Prescribed DDD of proton pump inhibitors from 1993 to 2022. Taken from the drug prescription reports from 1995 to 2023—pantoprazole (blue). Blue arrows refer to a change in the prescription of pantoprazole

Pantoprazole was introduced in 1994 as an analog of omeprazole, the first proton pump inhibitor. Omeprazole was introduced in 1989, and 13.1 million daily doses were prescribed in 1994, presumably because a study using ranitidine and omeprazole in patients with gastroesophageal reflux showed that omeprazole led to a faster cure of reflux esophagitis (Dammann et al 1986). Pantoprazole saw a sharp increase in prescriptions in the year following its introduction, presumably due to its lower DDD cost, and had more prescribed DDDs than omeprazole in 1995. In the following years, prescriptions for pantoprazole (Fig. 3) continued to rise, presumably due to its proven efficacy in the eradication treatment and short- and long-term therapy of reflux esophagitis (Schwabe and Paffrath 1996). This shows that positive clinical studies can lead to an increase in prescriptions.

With the introduction of omeprazole generics in 1999, the DDD costs of omeprazole fell sharply, most likely leading to the increase in prescriptions of omeprazole and a fall in prescriptions of pantoprazole, again demonstrating the impact of generic introduction on prescriptions. However, prescriptions of pantoprazole also increased again from 2001, which can presumably be explained by the supposed lack of interaction. However, this was refuted in 2002 by a meta-analysis in which pantoprazole interacted significantly more frequently with vitamin K antagonists than omeprazole (Labenz et al. 2002). Nevertheless, pantoprazole had an increase in prescriptions until 2003 despite higher DDD costs. As a result of the fixing of reference prices in the 2004 SHI Modernization Act, which were set for pantoprazole due to the lack of additional benefit compared to omeprazole, the DDD costs of pantoprazole fell in 2004 and 2005, which led to a further increase in prescriptions. This shows that the fixing of reference prices can lead to an increase in prescriptions, as the DDD costs are reduced.

In 2006, a statement was published by the German Medical Commission (AdkÄ) that the cheapest proton pump inhibitor should be preferred due to therapeutic equivalence (Statement AkdÄ, 2006). This almost certainly led to a drop in pantoprazole prescriptions in the following year, as omeprazole recorded an increase in prescriptions due to the lower DDD costs, which illustrates that a statement by the AkdÄ can also influence the prescribing behavior of physicians. However, after the drop in pantoprazole prescriptions in 2006 and 2007, pantoprazole prescriptions rose again in 2008.

Following the introduction of generic pantoprazole in 2009, prescriptions for pantoprazole rose sharply, while prescriptions for omeprazole stagnated, again demonstrating the effect of generic introduction. From 2010 to 2016, prescriptions of pantoprazole rose constantly, which was due to the lower DDD costs in contrast to omeprazole on the one hand and the supposed lack of interaction on the other.

In 2016, a review paper on proton pump inhibitors was published that criticized the use of proton pump inhibitors that was not appropriate for the indication (Mössner 2016). This is probably the cause of the decline in pantoprazole prescribing in 2017 and 2018, which shows that a negative analysis can lead to a decline in prescribing. However, here too, it depends on how much attention this analysis receives. From 2019, however, pantoprazole prescriptions increased again, possibly due to a review from 2018, which described that proton pump inhibitors are safer than expected (Koop 2018).

In 2022, the increase in prescriptions of pantoprazole leveled off again somewhat, which was probably due to a discussion about the excessive use of proton pump inhibitors triggered by a study from France (Lassalle et al. 2020). This is an example of how a discussion about the use of a medicine can lead to a change in prescribing behavior.

### Calcium channel blockers

**Fig. 4:**
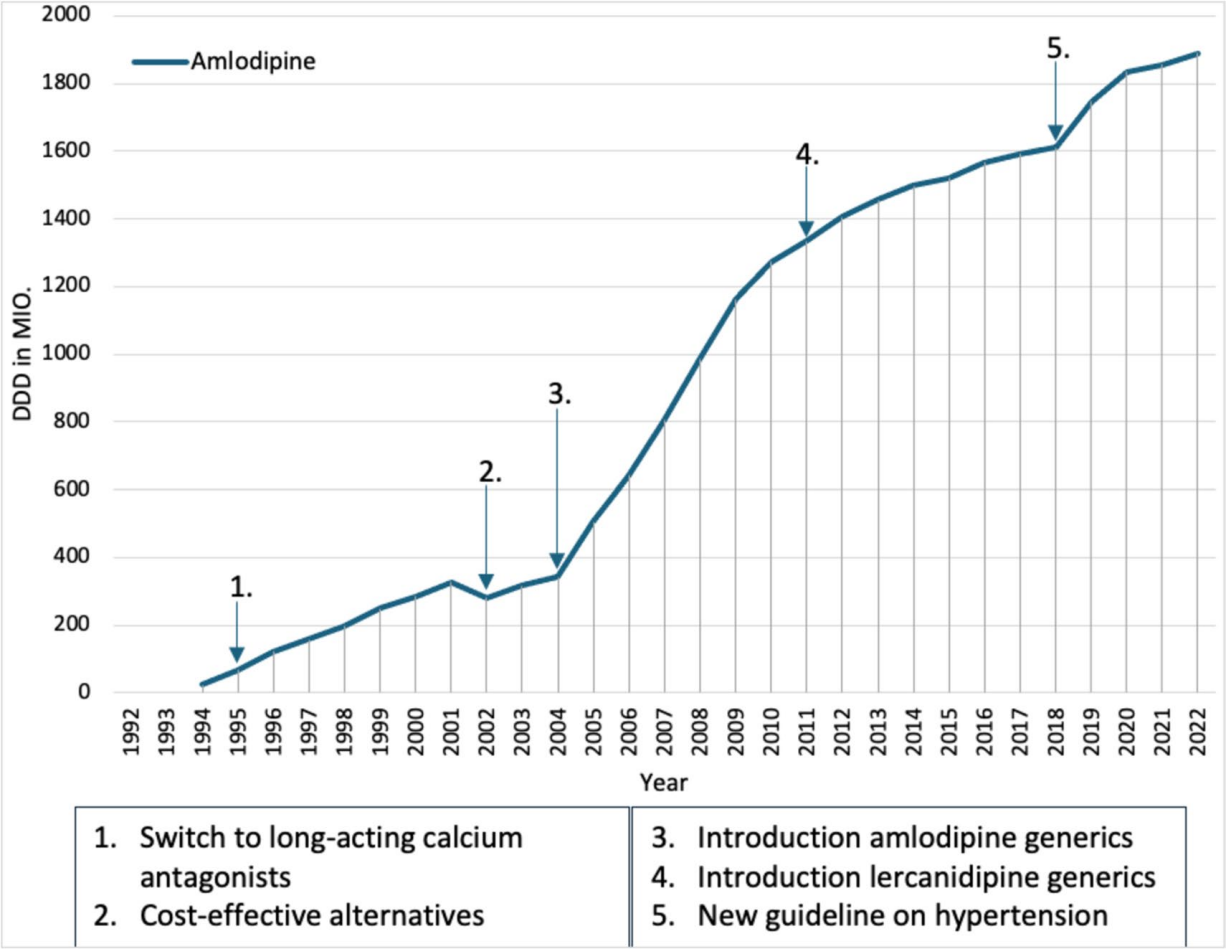
Prescribed DDD of calcium antagonists from 1992 to 2022. Taken from the drug prescription reports from 1994 to 2023—amlodipine (blue). Blue arrows refer to a change in the prescription of amlodipine

Amlodipine was introduced in Germany in May 1993 and recorded a sharp increase in prescriptions until 2001, probably mainly because amlodipine is a long-acting calcium antagonist (Fig. 4). Since 1994, the prescribing trend has been mainly towards long-acting calcium channel blockers, as a meta-analysis showed increased mortality from coronary heart disease and hypertension for the short-acting dihydropyridines (Furberg et al. 1995). This shows that a negative study for a competitor medicine can lead to an increase in prescribing.

In 2002, there was a drop in amlodipine prescriptions for the first time, presumably because the long-acting calcium antagonists nitrendipine and felodipine gained market share due to their generics and the resulting lower DDD costs (Schwabe and Paffrath 2003). In addition, prescriptions of cheaper analog preparations increased in 2002, which probably further increased the drop in prescriptions of amlodipine. This shows that the introduction of a generic version of a competitor medicine or the introduction of a cheaper competitor medicine can lead to a drop in prescriptions.

In 2003, however, prescriptions of amlodipine rose again, the exact cause of which is unclear. In 2004, generics for amlodipine were introduced, which only led to a sharp increase in prescriptions the following year due to the SHI Modernization Act. In addition, a study was published in 2004 in which amlodipine reduced cardiovascular events in coronary heart disease more than enalapril with the same tolerability (Nissen et al. 2004). These are also examples that show that the introduction of a generic medicine or a positive clinical trial can lead to an increase in prescriptions, although the increase in prescriptions here was probably primarily caused by the introduction of the generic medicine. The increase in prescriptions continued over the next few years, but leveled off after 2009, presumably since most patients had already switched from short-acting to long-acting calcium channel blockers.

Due to the introduction of lercanidipine generics in 2011, the increase in prescriptions of amlodipine leveled off further from 2012, which was probably due to the convergence of the DDD costs of lercanidipine and amlodipine. In addition, a meta-analysis comparing lercanidipine with amlodipine and other calcium channel blockers showed that lercanidipine led to less peripheral edema (Makarounas-Kirchmann et al. 2009). This again shows the effect of a positive study and the generic introduction of a competitor medicine. In the following years, prescriptions for amlodipine continued to rise, and in 2019, amlodipine again recorded a sharp increase in prescriptions, which is probably also due to the new European guideline for the treatment of hypertension (Williams et al. 2018). This again shows that a change in the guideline can lead to a sharp increase in prescriptions. In 2022, amlodipine was the most prescribed calcium channel blocker with almost 1900 million DDD, which was probably mainly due to the low DDD costs and the good study situation.

### HMG-CoA-reductase inhibitors

**Fig. 5:**
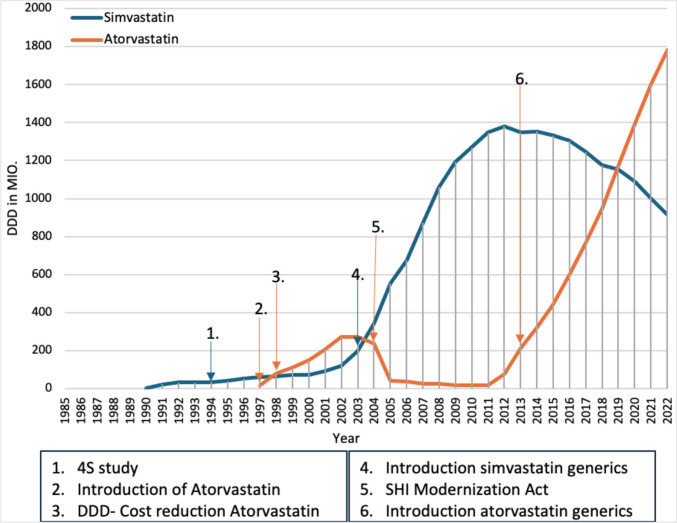
Prescribed DDD of HMG CoA reductase inhibitors from 1985 to 2022. Taken from the drug prescription reports from 1991 to 2023—atorvastatin (orange) and simvastatin (blue). Blue arrows refer to a change in the prescription of simvastatin. Orange arrows refer to a change in the prescription of atorvastatin

In 1990, two preparations with simvastatin were introduced as analog preparations of lovastatin. Presumably due to its lower DDD costs than lovastatin, simvastatin saw a sharp increase in prescriptions in the first few years after its introduction (Fig. 5). In 1993, however, prescriptions stagnated, although DDDs from the former GDR were included for the first time. This is possibly due to the drug budget of the SHI structural law. Between 1994 and 1997, prescriptions for simvastatin increased again, probably because a study in which 4444 patients with angina pectoris or previous myocardial infarction and a serum cholesterol level of 5.5–8.0 mmol/L were treated with either placebo or simvastatin showed a reduction in coronary deaths with simvastatin (Scandinavian Simvastatin Survival Study Group 1994). This is another example of how a positive clinical trial can lead to an increase in prescribing.

In 1997, atorvastatin was introduced, which, probably due to the expectation of greater benefit, had a sharp increase in prescriptions in the year of introduction, despite higher DDD costs. The expectation of greater benefit is based on a study in which atorvastatin at a dose of 10 mg/day lowered plasma LDL cholesterol concentrations at least as effectively as simvastatin (20 mg/day), but significantly more than pravastatin (20 mg/day), lovastatin (40 mg/day), and fluvastatin (40 mg/day) (Jones et al. 1998). Meanwhile, prescriptions for simvastatin increased, but not as much as for atorvastatin. One possible cause for the increase in prescriptions of HMG-CoA reductase inhibitors is discussed in the Drug Prescription Report as being the switch from fibrates to HMG-CoA reductase inhibitors, as no study has been able to demonstrate a reduction in mortality with fibrates (Schwabe and Paffrath 1999). Most likely, the reduction in the DDD cost of atorvastatin combined with the expectation of greater benefit led to the DDD of atorvastatin overtaking the DDD of simvastatin as early as 1998. This demonstrates once again that doctors are trying to provide good therapy in the most cost-conscious way possible. While prescriptions for atorvastatin rose sharply until 2003, simvastatin only recorded a slight increase in prescriptions.

However, this changed in 2003 with the introduction of simvastatin generics. It is likely that simvastatin prescriptions increased so much due to the generic launch, while atorvastatin prescriptions stagnated, again highlighting the effect of generic launch on prescriptions. In addition, in 2004, the SHI Modernization Act sets a reference price for atorvastatin at the level of simvastatin, since as a patent-protected active ingredient, it has no additional benefit compared to simvastatin. The reference price for atorvastatin applied from January 1, 2005, but the manufacturer decided not to adjust the DDD costs to the reference price level due to various factors (Schwabe and Paffrath 2006), which almost certainly led to the sharp drop in prescriptions of atorvastatin in 2005. This clearly shows that reference prices can also lead to a drop in prescriptions if the DDD costs are not adjusted to the reference prices.

Prescriptions for simvastatin continued to rise constantly until 2012, presumably due to the introduction of generics and the drop in prescriptions for atorvastatin. Presumably, the introduction of generic atorvastatin in 2012 led to the decline in prescriptions for simvastatin from 2013 onwards, while prescriptions for atorvastatin rose sharply. Here, too, the usual prescription behavior after a generic launch was evident. In 2019, the prescribed DDD of atorvastatin overtook the prescribed DDD of simvastatin and continued to rise, while prescriptions of simvastatin continued to fall. The reasons for the increase in prescriptions for atorvastatin are probably primarily a greater reduction in LDL cholesterol per mg of the drug (Jones et al. 1998), as well as the lower DDD costs.

### Levothyroxine

**Fig. 6:**
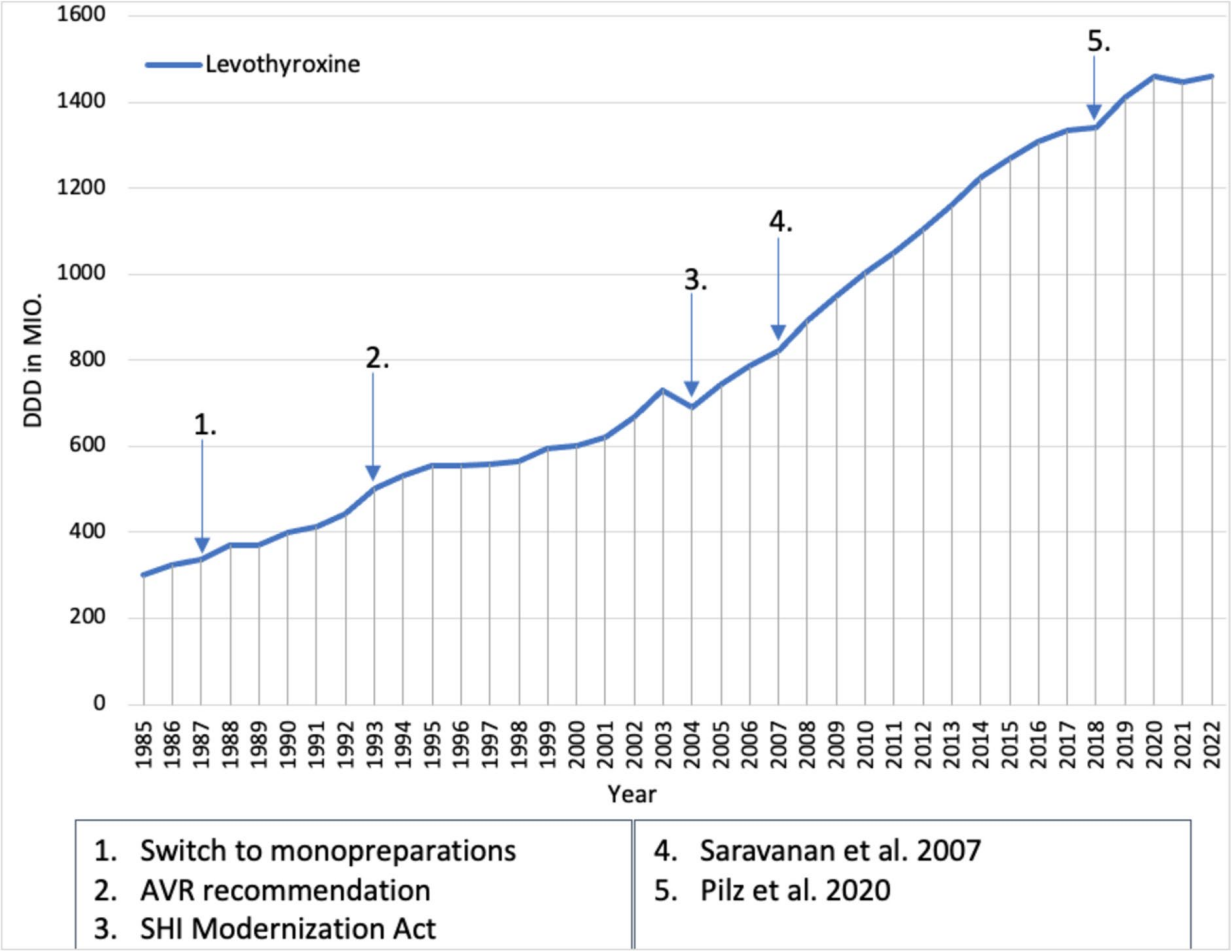
Prescribed DDD of levothyroxine (blue) from 1985 to 2022. Taken from the drug prescription reports from 1986 to 2023. Blue arrows refer to a change in the prescription of levothyroxine

Levothyroxine is used to treat hypothyroidism and was already one of the 30 most frequently prescribed medicines in 1985 with 450 million DDD (Fig. 6). After 1985, prescriptions of levothyroxine monopreparations rose sharply. As a possible reason for this, the AVR states that pharmacological therapy recommendations have presumably prevailed, as the combination preparations of liothyronine and levothyroxine lead to undesirable T3 peaks and should therefore no longer be used (Schwabe and Paffrath 1986). In addition, the DDD costs of levothyroxine monopreparations are lower than those of the combination preparations, which may also have contributed to the increase in prescriptions. The 1993 AVR stated that the prescription of thyroid therapeutics was still too low in relation to the frequency of illness (Schwabe and Paffrath 1993), which presumably led to a further increase in the prescription of levothyroxine.

Between 1996 and 1998, the increase in prescriptions of levothyroxine stagnated, but from 1999, prescriptions rose again. The exact reasons for this are unclear. The SHI Modernization Act and the associated increase in DDD costs probably led to the decline in prescriptions of levothyroxine in 2004. This is another example of the impact of the SHI Modernization Act on the prescriptions. However, the drop in prescriptions was already made up again in 2005.

In the following years, prescriptions for levothyroxine continued to rise sharply, which is presumably due to the decline in combination preparations, as it was again shown that these cause more undesirable peaks in triiodothyronine levels than monopreparations (Saravanan et al. 2007). In addition, normal T3 and T4 levels can be achieved with levothyroxine alone (Jonklaas et al. 2008). This demonstrates that a negative clinical trial for one medicine can lead to an increase in prescriptions for other medicines used in the same indication. Levothyroxine prescriptions rose steadily from 2005 to 2017, despite a debate that overdose with levothyroxine therapy may be more common than suspected and that levothyroxine is also frequently used in patients who do not need it (Taylor et al. 2014).

Levothyroxine had only a slight increase in prescriptions in 2018, but prescriptions rose sharply again in 2019 and 2020. In 2021, there was a slight decrease in prescriptions of levothyroxine, which may be due to a review article from 2020, as this questioned the use of levothyroxine in latent subclinical hypothyroidism (Pilz et al. 2020). This is another example of how the discussion about the use of a medicine leads to a stagnation in prescriptions. Levothyroxine was probably used in around 4 million patients with subclinical hypothyroidism in 2022 (Ludwig et al. 2023), although this would probably not be necessary for a large proportion of them. One reason for this is that, in recent years, the TSH threshold value above which levothyroxine is given for latent, subclinical hypothyroidism has continued to fall (Eckert 2013). Presumably, this treatment of the laboratory values, in which the patients usually had no clinical symptoms of hypothyroidism, contributed to the constant increase in the prescription of levothyroxine (Ludwig et al. 2023). In 2022, prescriptions of levothyroxine rose again, although the problem of misuse of levothyroxine is also becoming increasingly known. Levothyroxine is often used for weight loss or to mistreat depression, which is probably another reason for the constant increase in prescriptions (Topliss and Soh 2013; Vardarli et al. 2022; Persani et al. 2023).

### Torasemide

**Fig. 7:**
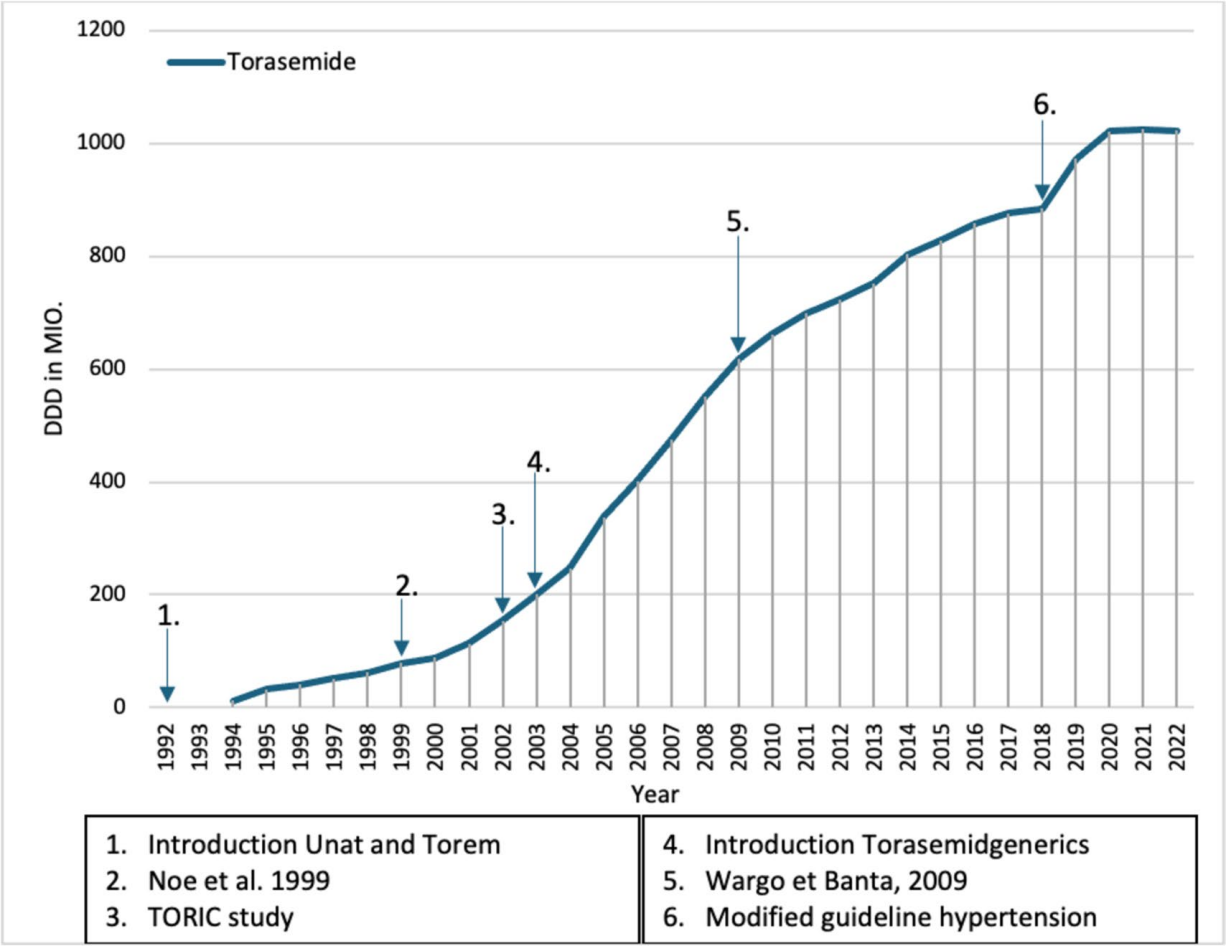
Prescribed DDD of torasemide (blue) from 1985 to 2022. Taken from the drug prescription reports from 1993 to 2023. Blue arrows refer to a change in the prescription of torasemide

Torem and Unat were introduced in 1992 and contain the drug torasemide, which has similar effects to furosemide and is therefore also a loop diuretic. In 1994, torasemide was among the 2000 most prescribed medicines for the first time (Fig. 7) and despite the higher DDD costs in contrast to furosemide, the prescriptions of torasemide increased steadily over the next few years. This was probably due to the longer-acting and slower onset of action compared to furosemide. The high and increasing prescription volume of loop diuretics is probably due to the high-dose dosage forms for patients with renal insufficiency.

Despite a 1999 study showing no differences in quality of life with torasemide compared to furosemide (Noe et al. 1999), prescriptions of torasemide continued to rise despite the higher DDD costs. This is probably due more to the considerable advertising effort than to a uniform data situation (Schwabe and Paffrath 2002), which shows that not every negative clinical study necessarily leads to a drop in prescriptions. It is also possible that some doctors have had good experiences with torasemide and therefore prefer it. Presumably, the publication and promotion of the TORIC study in 2002, which showed the superiority of torasemide, led to the further increase in prescriptions of torasemide in 2003, again demonstrating that a positive study can lead to an increase in prescribing. In the TORIC study, heart failure patients were treated with either torasemide, furosemide, or other diuretics for 12 months in addition to their existing heart failure therapy. Mortality and morbidity were significantly lower in the torasemide group than in the furosemide group (Cosín and Díez 2003). However, it should be noted that this study is not conclusive due to structural weaknesses, as the data from two arms of a non-randomized, open observational study were analyzed in such a way as to give the impression of a true randomized study (Schwabe and Paffrath 2003).

Torasemide generics were also introduced in December 2003, which almost certainly led to the sharp increase in DDDs in 2004. Introduction of generics can lead to an increase in prescriptions. Despite still being more expensive than furosemide, prescriptions of torasemide continued to rise over the next few years, presumably because, contrary to the study by Noe et al. 1999, it has a difference on quality of life. In 2009, more recent reviews (Wargo and Banta 2009) repeatedly attempted to highlight a presumed advantage of torasemide over furosemide, but other than the slightly more favorable pharmacokinetics of torasemide compared to furosemide, no convincing clinical studies have been published demonstrating pharmacodynamic superiority of torasemide (Schwabe and Paffrath 2010). Nevertheless, prescriptions of torasemide continued to rise slightly until 2018.

In 2019, the loop diuretics, which had previously had constant prescription figures for years due to the decrease in prescriptions of furosemide and increase in prescriptions of torasemide, had an increase in prescriptions. This is most likely due to the new European guideline on the treatment of hypertension (Williams et al 2018). The increase in prescriptions also continued in 2020. This demonstrates once again that prescriptions can increase sharply following a change in the guideline in favor of a medicine. In 2021, the increase in prescriptions stagnated, and in 2022, prescriptions of torasemide fell for the first time since its introduction. The drop in prescriptions was possibly since some patients who were started on torasemide for hypertension therapy in 2019 or whose dose was increased did not tolerate it and were therefore switched to another medication for hypertension therapy.

### β-Adrenoceptor antagonists

**Fig. 8:**
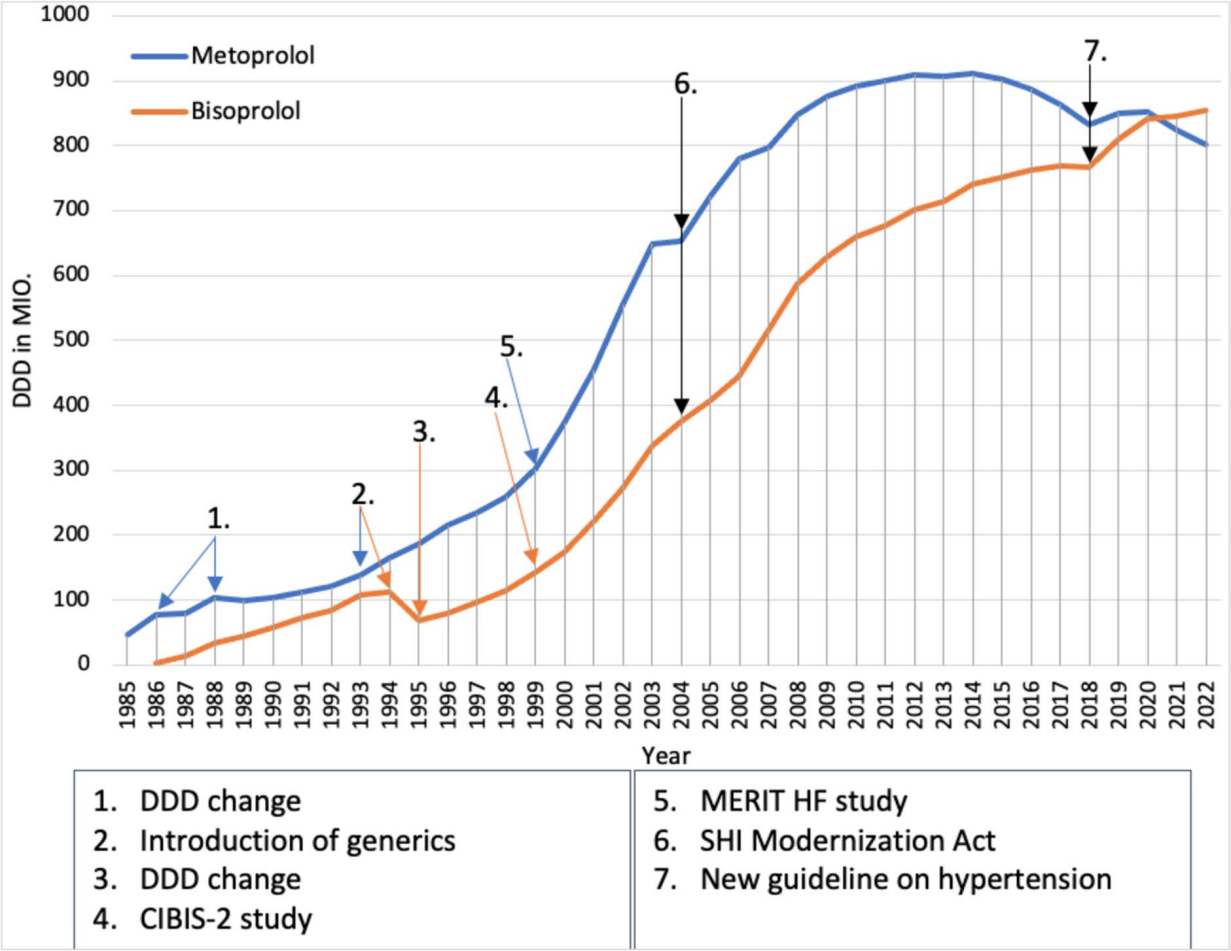
Prescribed DDD of beta-adrenoceptor antagonists from 1985 to 2022. Taken from the drug prescription reports from 1993 to 2023—metoprolol (blue) and bisoprolol (orange). Blue arrows refer to a change in the prescription of metoprolol or DDD change. Orange arrows refer to a change in the prescription of bisoprolol or a DDD change. Black arrows refer to a change in the prescription of metoprolol and bisoprolol

Figure 8 shows the prescription trends for metoprolol and bisoprolol from 1985 to 2022. In 1985, there were three pharmaceutical preparations containing the medicine metoprolol. The DDDs of metoprolol increased on average until 1993, even if the individual preparations developed in opposite directions in some cases. In 1986 and 1988, there was a change in the DDD in each year, so the sharp increases in the DDD of metoprolol were not real. In addition, bisoprolol was introduced in 1986, which was frequently prescribed in 1987, presumably due to its lower DDD costs, leading to a stagnation in the increase of metoprolol prescriptions. Bisoprolol prescriptions also continued to rise over the next few years. The AVR discusses the switch from non-selective receptor antagonists to selective receptor antagonists as a possible reason for the increase in prescriptions of beta1-selective receptor antagonists (Schwabe and Paffrath 1991). In addition, the prescriptions of the former GDR were included from 1993 onwards.

Furthermore, new generics of metoprolol were introduced in 1993 that were even cheaper than bisoprolol, which very likely led to the increase in prescriptions for metoprolol and a stagnation in prescriptions for bisoprolol. In October 1994, the patent for bisoprolol (Concor) expired, which presumably led to bisoprolol being prescribed more frequently again. However, this is difficult to see in the graph due to a change in the DDD for bisoprolol in 1995. Metoprolol and bisoprolol also show that the introduction of a generic medicine generally leads to an increase in prescriptions.

The prescriptions for bisoprolol and metoprolol also continued to rise in the following years. In addition, the publication of the CIBIS-2 study in 1999 led to a sharp increase in the prescription of bisoprolol. In the CIBIS II study, patients with heart failure NYHA 3 or 4 and a left ventricular ejection fraction of 35% or less received either a placebo or bisoprolol in addition to standard therapy with an ACE inhibitor and a diuretic. The CIBIS-II study was terminated early after an interim analysis because significantly fewer sudden deaths occurred in patients receiving bisoprolol than in patients receiving placebo. The treatment effects were independent of the severity or cause of heart failure (CIBIS II study 1999). Metoprolol also saw a strong increase in prescriptions in 1999 due to the publication of a study, the MERIT HF study. In the MERIT HF study, patients with heart failure NYHA 2-4 and a left ventricular ejection fraction of 40% or less were treated with either metoprolol or placebo in addition to standard therapy. This study was also terminated prematurely on the recommendation of the independent safety committee, as there were fewer sudden deaths and fewer deaths due to worsening heart failure in the metoprolol group than in the placebo group (MERIT-HF study 1999). It is unclear why the prescriptions of metoprolol increased more than the prescriptions of bisoprolol, as bisoprolol would have been the better choice due to its lower DDD costs. Nevertheless, it is another good example of how a positive clinical study can lead to a sharp increase in prescribing.

In 2004, the increase in DDD prescriptions for metoprolol stagnated for the first time since 1989, and the DDD for bisoprolol also rose less sharply than in previous years. This is presumably due to the SHI Modernization Act. However, it is unclear why bisoprolol and especially metoprolol recorded a lower increase in prescriptions due to the SHI Modernization Act, as both medicines even showed a slight decrease in DDD costs in 2004. After 2005, prescriptions of bisoprolol and metoprolol increased again, but it was not until 2007 that the increase in prescriptions of bisoprolol was greater than that of metoprolol. The AVR cites the more favorable DDD costs, the better pharmacokinetic profile as well as the higher oral bioavailability and the lower dependence on CYP2D6 metabolism as possible reasons for this (Schwabe and Paffrath 2006).

From 2009, bisoprolol and metoprolol have seen a lower increase in prescriptions than in previous years. This is presumably due to the discussion about hypertension treatment with metoprolol and bisoprolol. Here too, as with pantoprazole, the discussion about the use of a medicine can lead to a stagnation in prescription growth. From 2011, the increase in prescriptions of metoprolol then stagnated completely and from 2015, prescriptions of metoprolol fell in favor of bisoprolol. However, bisoprolol prescriptions increased only slightly and from 2015 the increase in bisoprolol prescriptions also stagnated, which is probably due to the renewed debate about the use of beta-adrenoceptor antagonists in stage 1 of hypertension therapy (DiNicolantonio et al. 2015).

In 2018, prescriptions for bisoprolol declined for the first time, probably because beta-adrenoceptor antagonists are no longer listed as first-line agents in the American guidelines, except for special indications such as coronary heart disease or heart failure (Whelton et al. 2018). This shows that a new guideline, in which a medicine is no longer the medicine of first choice, can lead to a decrease in prescriptions. Although it is an American guideline, some doctors may have thought that this would also be the case in the new European guideline. The reason for the increase in prescriptions of beta1-adrenoceptor antagonists in 2019 is unclear. However, it is possible that prescriptions for beta-adrenoceptor antagonists increased again in 2019, as the new European hypertension guideline does include them as first-line therapy for certain indications (Williams et al 2018). However, the increase in metoprolol prescriptions stagnated again in 2020 and then fell in favor of bisoprolol. Bisoprolol prescriptions continued to rise and replaced metoprolol as the leading active ingredient in 2021, which seems reasonable given its better pharmacokinetic profile (Ludwig et al. 2022).

### Ramipril

**Fig. 9:**
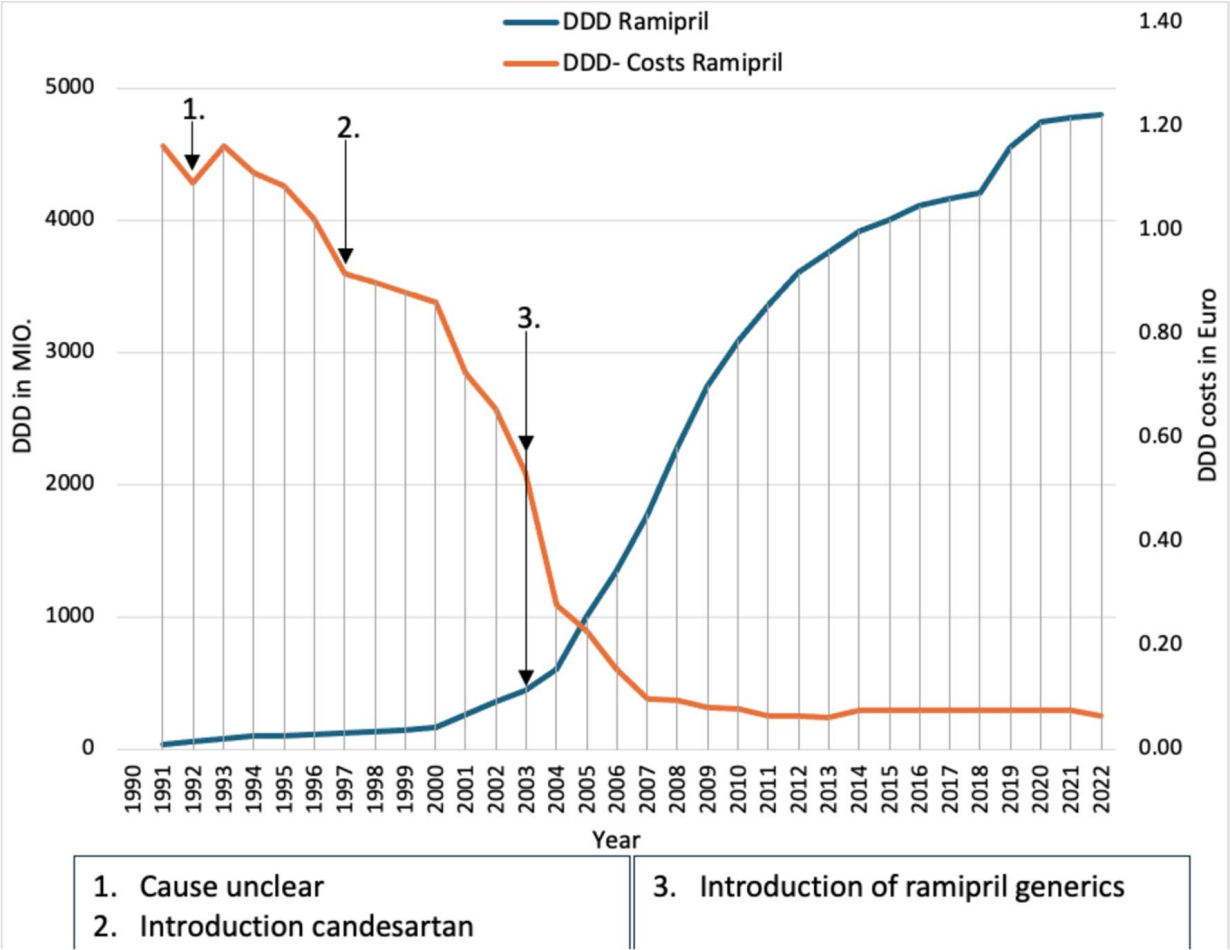
Comparison of the prescribed DDD and DDD costs of ramipril from 1985 to 2022—DDD of ramipril (blue) and DDD costs of ramipril (orange). Black arrows indicate a change in the prescribed DDD and the DDD costs

Ramipril was launched in October 1990 and was already among the top 2000 most frequently prescribed drugs in 1991, presumably because ramipril was the cheapest ACE inhibitor on the German pharmaceutical market with a DDD price of the equivalent of EUR 1.16 (Fig. 9). Although ramipril was the cheapest ACE inhibitor prescribed, it was still more expensive than most other antihypertensives, showing that physicians prescribe more expensive medicines when necessary for evidence-based therapy. This finding is particularly interesting as recent studies showed that DDD cost is the main driver for prescribing antibacterial drugs, leading to largely irrational (i.e., not scientifically justified) drug use in this area (Bindel and Seifert, 2024a, 2024b). The DDD costs continued to fall thereafter, which probably further stimulated prescriptions. From 1997 to 2000, the DDD cost decrease of ramipril stagnated, the exact reason for this is unclear, possibly because candesartan had a strong increase in prescriptions despite higher DDD costs. In 2002 and 2003, the DDD costs of ramipril continued to fall, which, together with the HOPE study, probably contributed to the increase in prescriptions of ramipril.

The generic launch of ramipril in November 2003 almost halved the average DDD costs in 2004, which most likely resulted in the sharp increase in prescriptions. This is a good illustration of how sharply the DDD costs fall after a generic launch and why the prescribed DDDs rise so sharply. DDD costs also continued to fall in the following years, which further boosted the increase in prescriptions. Since 2007, the average DDD cost of ramipril has been around 7 cents, making ramipril the cheapest ACE inhibitor, which, together with the wide range of approvals and studies, is responsible for the high number of DDDs prescribed. However, the real costs of ACE inhibitors are higher, as ramipril is most frequently prescribed at a higher daily dose (5 mg) than the WHO DDD (2.5 mg) (Ludwig et al. 2022).

### Candesartan

**Fig. 10:**
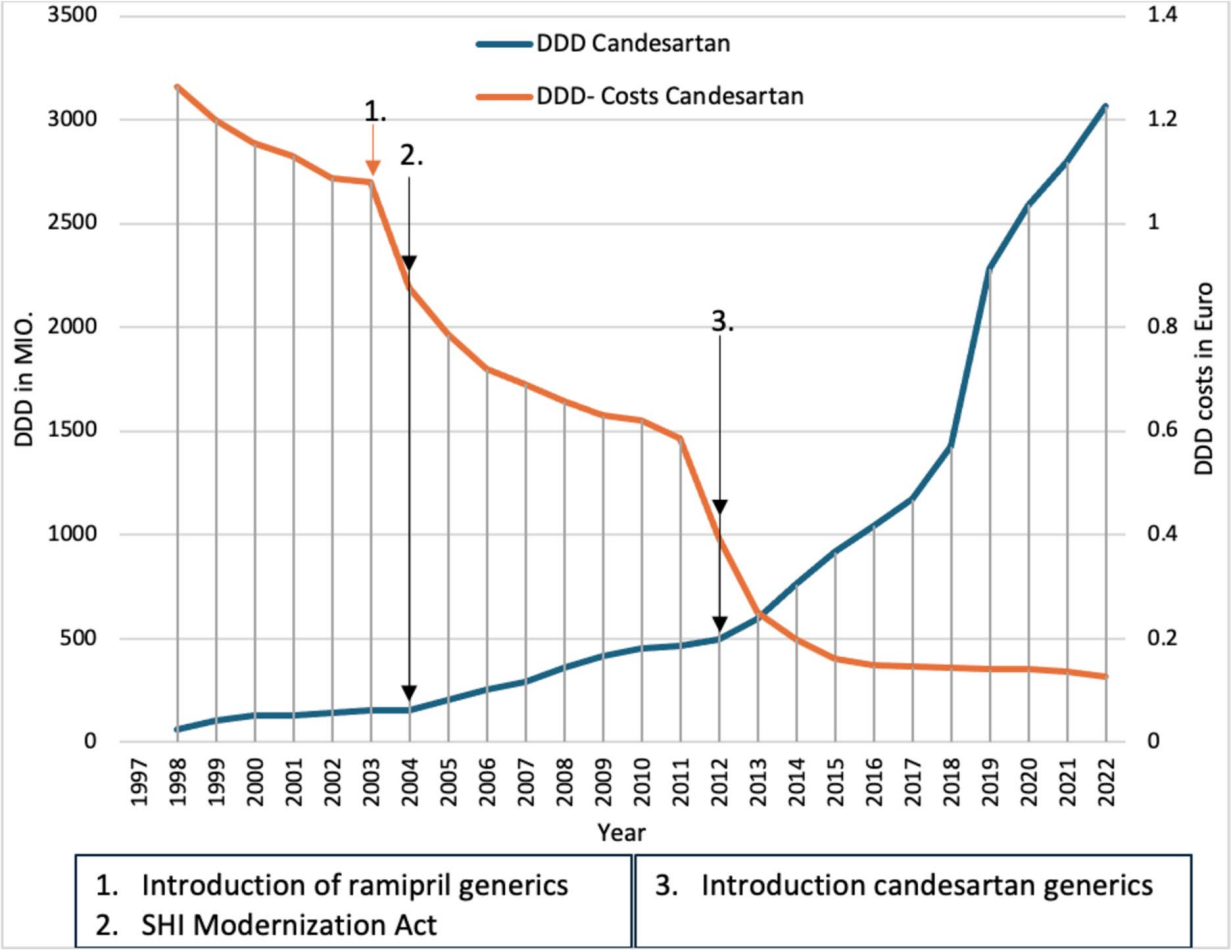
Comparison of the prescribed DDD and DDD costs of candesartan from 1985 to 2022—DDD of candesartan (blue) and DDD costs of candesartan (orange). Black arrows indicate a change in the prescribed DDD and the DDD costs

Candesartan was introduced in December 1997 and, in contrast to ramipril, had a strong increase in prescriptions despite higher DDD costs (Fig. 10). Presumably, the sharp drop in DDD costs in 2004 is mainly due to the SHI Modernization Act, which set a fixed price for candesartan. In addition, the introduction of ramipril generics probably also contributed to the drop in costs, as Candesartan wanted to retain market share. The decrease in DDD costs is probably the explanation for the increase in candesartan prescriptions from 2005 onwards.

In 2012, candesartan generics were introduced, which led to a drastic decrease in DDD costs and then most likely to a sharp increase in prescriptions. Since 2016, DDD costs have stagnated at a low level, but prescriptions have continued to rise due to other factors such as the valsartan recall. Although the DDD costs of candesartan are still higher than the DDD costs of ramipril, it should be noted that the actual DDD cost of ramipril is higher than the WHO DDD cost (Ludwig, Mühlbauer, and Seifert, 2022). Therefore, both medicines cost approximately the same.

### Pantoprazole

**Fig. 11:**
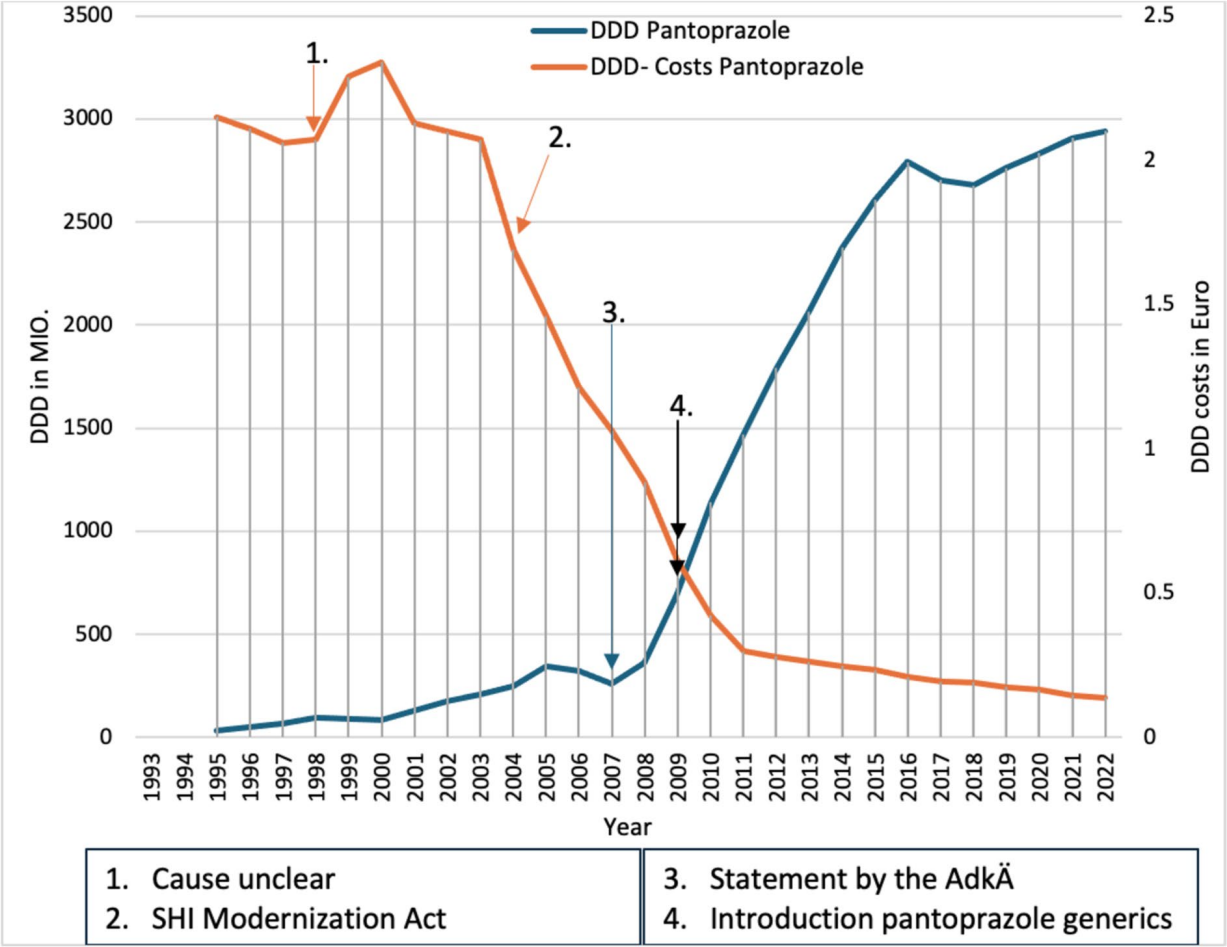
Comparison of the prescribed DDD and DDD costs of pantoprazole from 1985 to 2022—DDD of pantoprazole (blue) and DDD costs of pantoprazole (orange). Black arrows indicate a change in the prescribed DDD and the DDD costs

Pantoprazole was introduced in 1994 as an analog preparation of omeprazole and had a strong increase in prescriptions (Fig. 11), probably due to the lower DDD costs compared to omeprazole (40 Pfennigs cheaper in 1995). It is likely that the introduction of generic omeprazole in 1999 reduced the number of prescriptions for pantoprazole, but it is unclear why DDD costs increased as a result. This may be artificial, as fewer doses of pantoprazole are prescribed and these may then be dispensed in smaller packs, leading to higher weighted DDD costs. Due to the 2004 SHI Modernization Act and the fixed amounts that applied to pantoprazole from 01.01.2005, DDD costs fell in 2004 and 2005. The decline in DDD costs and the supposed lack of interaction with pantoprazole probably led to the increase in prescriptions in subsequent years. The DDD costs also continued to fall in 2007 and 2008, probably due to competition from the much cheaper omeprazole generics. The drop in prescriptions for pantoprazole in 2007 despite a reduction in costs is certainly due to a statement by the German Medical Commission.

With the introduction of pantoprazole generics in 2009, the DDD costs of pantoprazole once again fell sharply. In 2009, pantoprazole was cheaper than omeprazole for the first time since 1999 (7 cents per DDD), which was most likely the cause pantoprazole saw a sharp increase in prescriptions. The DDD costs continued to fall over the next few years, which further stimulated the increase in prescriptions. The constant increase in prescriptions can therefore presumably be attributed to the lower DDD costs in contrast to other proton pump inhibitors and the supposed lack of interaction.

### Cause for changes in prescription

Tables 2, 3, 4 and 5 show how often an event leads to a change in prescription and the extent of this change. However, it should be noted that these calculations are also influenced by other factors and should therefore only be considered in the overall context.

## Limitations

This retrospective analysis from 1985 to 2022 focuses only on the top 10 medicines in 2022, which is why significant changes in the treatment of individual diseases are difficult to depict. In addition, only a few causes for a decline in prescriptions can be identified for the top 10 medicines in 2022, as these medicines are currently the most frequently prescribed and therefore have not seen a sharp decline in prescriptions.

There were often several events in a short period of time, so it was sometimes somewhat unclear which event had mainly caused the change in prescription and what part the other event played in the change in prescription.

The prevalence of the diseases that are treated with the medicines mentioned may also have changed and are therefore another possible reason for a change in prescribing. This is difficult to control without a large study in Germany. It is also possible that the prescription stagnation of a medicine may have been caused by a saturation of the market if the incidence of the disease has remained the same and all patients are well controlled with a medicine.

The conclusions drawn from the retrospective analysis of prescription changes and causes are never 100% certain. Statistical or economic tests would have to be carried out to analyze one cause at a time. In addition, prospective studies and surveys among physicians regarding their prescription behavior are needed.

Due to the lack of DDD values for bisoprolol 1995 and metoprolol 1985 and 1987, this could not be converted to the current DDD. Therefore, the prescription changes that followed events in these years cannot be shown correctly in Fig. 9.

## Conclusions

The main reasons for an increase in prescriptions are the introduction of generics and positive clinical studies. An extension of the indication also often leads to an increase in prescriptions, as significantly more patients can then be treated with this medicine. In addition, a negative clinical trial for a competing medicine can lead to an increase in prescriptions. Other reasons for an increase in prescriptions are a change in the guideline in favor of a medicine and the treatment of laboratory values without clinical symptoms.

The main reasons for a drop in prescriptions are the introduction of new analog preparations or medicines that are used for the same indication. The introduction of generics of competitor medicines also often leads to a drop in prescriptions. The SHI Modernization Act of 2004 also led to a decrease in prescriptions for some medicines, on the one hand, due to the increased co-payment for medicines, and on the other hand, due to the non-adjustment of DDD costs to a fixed amount. A negative clinical study also often leads to a decline in prescriptions. Another possible reason for a decline in prescriptions is a new guideline in which the medicine is no longer the medicine of choice.

After the introduction of a medicine, the DDD costs usually only decrease. This is usually due to competition from other medicines used for the same indication. As doctors usually prescribe the cheaper medicine, this often leads to a decrease in DDD costs. Another reason for the decline in DDD costs is the introduction of generics. Fixed amounts, as in the 2004 SHI Modernization Act, also lead to a reduction in DDD costs.

To illustrate significant changes in the treatment of certain diseases, the current top 10 should be compared with the top 10 from 1985. This would make it possible to identify the causes of a drop in prescriptions. It would also be interesting to see how the other medicines in the top 30 medicines of 2022 have developed, as these are presumably medicines that were once in the top 10 or medicines that are likely to be in the top 10 most frequently prescribed medicines in the coming years.

In addition, similar studies in other countries over such a long period of time would be useful to analyze and compare the understudied cultural aspects of prescribing behavior and the influences of the healthcare system on the prescribing behavior of physicians.

## Take-home messages

- Causes for an increase in prescribing include a generic launch, an extension of the indication or the withdrawal of a competitor product (Table 2). A positive clinical study or, a drop in costs or a change in guidelines in favor of the medicine also lead to an increase in prescriptions (Table 3). Irrational factors play a role as well.
- The reasons for a drop in prescriptions are the introduction of a generic version of a competitor product, a negative clinical study, the SHI Modernization Act, or a new guideline that excludes the medicine (Tables 4 and 5).
- The costs of a medicine usually fall constantly after the medicine introduction, which is due to competition with other medicines and laws that attempt to reduce the DDD costs. The expiry of a patent and the introduction of low-cost generics also cause DDD costs to continue to fall sharply.
- This is the most comprehensive long-term analysis of drug prescriptions in Germany. To this end, the AVR considered only a 10-year period and did not consider developments beyond a decade systematically because of the type of analysis chosen.

**Table 2:** Influences on the DDD of the medicines due to the introduction of generics, an extension of the indication or the withdrawal of a competitor product. The change in prescriptions is calculated as the difference between the percentage change 2 years before the event and the percentage change in the event year and the following year. For reasons of clarity, only medicine-specific indication extensions were considered; if only one medicine received this indication extension, it was not taken into account. Decreases are in bold. Falsified calculations due to DDD changes are italicized

Medicine	Year generics launched		Year indication extension		Year of withdrawal of a competitor product	
		Difference percentage change		Difference percentage change		Difference percentage change
Ramipril	2003	**− 39.45%**	2002	**− 9%**		
Candesartan	2012	17.29%			2018	67.34%
Metoprolol	1993	20.07%	1999	23%		
Bisoprolol	1994	*− 84.26%*	1999	5%		
Amlodipine	2004	63%				
Pantoprazole	2009	204.83%				
Simvastatin	2003	126.35%				
Atorvastatin	2012	1176.16%				
Torasemide	2003	**− 15.31%**				
Levothyroxine-natrium

**Table 3:** The effects on the DDD of the medicines due to a change in a guideline in favor of the medicine, a positive clinical study, and a negative clinical study for a competitor medicine with the same indication as the medicine. The change in prescriptions is calculated as the difference between the percentage change 2 years before the event and the percentage change in the event year and the following year. Here too, for reasons of clarity, a positive clinical study for the medicine and a negative clinical study for a competitor medicine were limited in each case. Decreases are in bold

Medicine	Year of the amended guideline in favor of the medicine		Year of a positive clinical study		Year of a negative clinical study for a competitor product	
		Difference percentage change		Difference percentage change		Difference percentage change
Ramipril	2018	5.47%	2000	57%		
Candesartan	2018	67.16%	2018	67.16%		
Metoprolol			1999	23%		
Bisoprolol			1999	4%		
Amlodipine	2018	5%	2004	62.65%	1995	420.34%
Pantoprazole			2018	4.91%		
Simvastatin			1994	**− 19.65%**		
Atorvastatin			1998	476.74%		
Torasemide	2018	5.39%	2002	32.67%		
Levothyroxine-natrium			2008	4.37%	2007	0.17%

**Table 4:** The impact on the DDD of the medicines due to the introduction (or the introduction of generics) of a competitor product or a negative clinical study. The change in prescriptions is calculated as the difference between the percentage change 2 years before the event and the percentage change in the event year and the following year. For reasons of clarity, only one example was given for each event. These events often only lead to a reduced increase in prescriptions compared to the previous year or that another event in the previous year triggered a sharp increase in prescriptions, so the entire course should be considered. Decreases are in bold. Falsified calculations due to DDD changes are italicized

Medicine	Year generic launch/new launch of competitor product		Year of the negative clinical study	
		Difference percentage change		Difference percentage change
Ramipril	1997	**7.84%**		
Candesartan	2003	**− 3.12%**		
Metoprolol	1994	13.28%	2015	**− 3%**
Bisoprolol	1993	*− 12.81%*	2015	**− 3%**
Amlodipine	2001	**− 27%**	2002	
Pantoprazole	1999	**− 98.46%**	2016	**− 22.40%**
Simvastatin	1997	**− 18.48%**		
Atorvastatin	2003	**− 81.58%**		
Torasemide			1999	**− 12.1%**
Levothyroxine-natrium

**Table 5:** The the influences on the DDD of the medicines due to the SHI Modernization Act and a changed guideline that excludes the medicine. The change in prescriptions is calculated as the difference between the percentage change 2 years before the event and the percentage change in the event year and the following year. Decreases are in bold

Medicine	SHI Modernization Act 2004		Year of the amended guideline against a medicine	
		Difference percentage change		Difference percentage change
Ramipril	2004	52.67%		
Candesartan	2004	12.11%		
Metoprolol	2004	**− 31.10%**	2017	− 4%
Bisoprolol	2004	**− 30.98%**	2017	− 2%
Amlodipine	2004	63%		
Pantoprazole	2004	4.17%		
Simvastatin	2004	59.90%		
Atorvastatin	2004	**− 115.31%**		
Torasemide	2004	**− 7.28%**		
Levothyroxine-natrium	2004	**− 15.97%**		

## Data Availability

All source data for this study are available upon reasonable request from the authors.

## Abbreviations

SHI: Statutory health insurance
GDR: German Democratic Republic
AVR: Arzneiverordnungsreport (Drug Prescription Report)
AdkÄ: Arzneimittelkommission der deutschen Ärzteschaft (Drug Commission of the German Medical Association)
DDD: Daily defined dose (tägliche Tagesdosis)
WHO: World Health Organization
ACE: Angiotensin-converting enzyme
AT1R: Angiotensin II receptor type 1

## References

[CR1] BfArM (2018) Valsartan: BfArM recommends switching to medicines not affected by the recall. Press release 6/18 from 13.07.2018

[CR2] Bindel LJ, Seifert R (2024a) Costs are a major driver of antibacterial drug prescriptions in Germany: market analysis from 1985 to 2022. Naunyn Schmiedebergs Arch Pharmacol. 10.1007/s00210-024-03171-y 10.1007/s00210-024-03171-y PMC11522090 38842562

[CR3] Bindel LJ, Seifert R (2024b) Daily defined dose costs have stronger influence on antibacterial drug prescriptions in Germany than bacterial resistance: economic factors are more important than scientific evidence. Naunyn Schmiedebergs Arch Pharmacol. 10.1007/s00210-024-03435-7 10.1007/s00210-024-03435-7 PMC11920358 39302420

[CR4] Blankart KE, Stargardt T (2020) The impact of drug quality ratings from health technology assessments on the adoption of new drugs by physicians in Germany. Health Economics 29(S1):63–82. 10.1002/hec.4108. lastaccessed06.09.2024 32542875 10.1002/hec.4108

[CR5] Blankart KE, Vandoros S (2024) Explaining why increases in generic use outpace decreases in brand name medicine use in multisource markets and the role of regulation. PLOS ONE 19(5):e0301716. 10.1371/journal.pone.0301716. lastaccessed06.09.2024 38696520 10.1371/journal.pone.0301716 PMC11065256

[CR6] Bundesbank (2024) Purchasing power equivalents of historical amounts in German currencies: https://www.bundesbank.de/de/statistiken/konjunktur-und-preise/-/kaufkraftaequivalente-historischer-betraege-in-deutschen-waehrungen-615162. last accessed: 29.08.2024

[CR7] Burt C (2002) National trends in use of medications in office- based practice from 1985 to 1999. 10.1377/hlthaff.21.4.206 10.1377/hlthaff.21.4.206 12117131

[CR8] Carney G, Kim JD, O’Sullivan C, Thompson W, Bassett K, Levin J, Dormuth CR (2022) Treatment pattern trends of medications for type 2 diabetes in British Columbia, Canada. BMJ Open Diabetes Res Care. https://pubmed.ncbi.nlm.nih.gov/36356988/10.1136/bmjdrc-2022-002995 PMC9660664 36356988

[CR9] CIBIS II Study (1999) The cardiac insufficiency bisoprolol study II (CIBIS II): a randomized trial. Lancet 353:9–13 10023943

[CR10] CONSENSUS Trial Study Group (1987) Effects of enalapril on mortality in severe congestive heart failure: results of the Cooperative North Scandinavian Enala-pril Survival Study (CONSENSUS). New Engl J Med 316:1429–1435 2883575 10.1056/NEJM198706043162301

[CR11] Cosin J, Diez J, TORIC investigators (2003) Torasemide in chronic heart failure: results of the TORIC study. Eur J Heart Fail 4: 507-513 10.1016/s1388-9842(02)00122-8 12167392

[CR12] Dammann, Blum, Lux, Rehner, Riecken, Schiessel, Wienbeck, Witzel (Editorial Board), Berger (Statistik) (1986) Differences in healing tendency of reflux oesophagitis with omeprazol and ranitidine. Results of an Austrian-German-Swiss multicenter trial. Dtsch Med Wochenschr 111(4):123-128. 10.1055/s-2008-1068412 10.1055/s-2008-1068412 3510847

[CR13] DiNIcolantonio JJ, Fares H, Niazi AK et al (2015) ß-Blockers in hypertension, diabetes, heart failure and acute myocardial infarction: a review of the literature. Open Heart 21(1):e000230 10.1136/openhrt-2014-000230 PMC4371808 25821584

[CR14] Eckert N (2013) Hypothyroidism - or not? When the thyroid gland needs thyroxine. https://deutsch.medscape.com/artikel/4901614, last accessed on 15.04.2024

[CR15] Furberg C, Psaty BM, Meyer JS (1995) Nifedipine. Dose-related increase in mortality in patients with coronary heart disease. Circulation 92:1326–1331 7648682 10.1161/01.cir.92.5.1326

[CR16] Jones P, Kafonek S, Laurora I, Hunninghake D for the CURVES Investigators (1998) Comparative dose efficacy study of atorvastatin versus simvastatin, pravastatin, lovastatin, and fluvastatin in patients with hypercholesterolemia (The Curves Study). Am J Cardio!' 81: 582-587 10.1016/s0002-9149(97)00965-x 9514454

[CR17] Jonklaas J, Davidson B, Bhagat S, Soldin SJ (2008) Triiodothyronine levels in athyreotic individuals during levothyroxine therapy. JAMA 299:769–777 18285588 10.1001/jama.299.7.769

[CR18] Koop H (2018) Prescribing practice and risks of proton pump blockers - fiction and fact? Z Gastroenterol 56:264–274 29341042 10.1055/s-0043-125340

[CR19] Labenz J, Petersen K-U, Rösch W, Koelz H (2002) Selection of proton pump inhibitors. Dtsch Ärtebl 99:C1940–C1944

[CR20] Lassalle M, Le Tri T, Bardou M et al (2020) Use of proton pump inhibitors in adults in France: a nationwide drug utilization study. Eur J Clin Pharmacol 76:449–457. 10.1007/s00228-019-02810-1 31838548 10.1007/s00228-019-02810-1

[CR21] Ludwig W, Mühlbauer B, Seifert R(2021) Drug prescription report 2021: Springer, Berlin/Heidelberg

[CR22] Ludwig W, Mühlbauer B, Seifert R (2022) Drug prescription report 2022: Springer, Berlin/Heidelberg

[CR23] Ludwig W, Mühlbauer B, Seifert R (2023) Drug prescription report 2023: Springer, Berlin/Heidelberg

[CR24] Makarounas-Kirchmann K, Glover-Koudounas S, Ferrari P (2009) Results of a metaanalysis comparing the tolerability of lercanidipine and other dihydropyridine calcium channel blockers. Clin Ther 31:1652–1663 19808126 10.1016/j.clinthera.2009.08.010

[CR25] Mancia G, Fagard R, Narkiewicz K, Redón J, Zanchetti A, Böhm M, Christiaens T, Cifkova R, De Backer G, Dominiczak A, Galderisi M, Grobbee DE, Jaarsma T, Kirchhof P, Kjeldsen SE, Laurent S, Manolis AJ, Nilsson PM, Ruilope LM, Schmieder RE, Sirnes PA, Sleight P, Viigimaa M, Waeber B, Zannad F (2013) Task Force Members (2013): 2013 ESH/ESC Guidelines for the management of arterial hypertension: the task force for the management of arterial hypertension of the European Society of Hypertension (ESH) and of the European Society of Cardiology (ESC). J Hypertens. 31(7):1281–357. 10.1097/01.hjh.0000431740.32696.cc 23817082 10.1097/01.hjh.0000431740.32696.cc

[CR26] Menzies-Gow AN, Tran TN, Stanley B, Carter VA, Smolen JS, Bourdin A, Fitzgerald JM, Raine T, Chapaneri J, Emmanuel B, Jackson DJ, Price DB (2024) Trends in systemic glucocorticoid utilization in the United Kingdom from 1990 to 2019: a population-based, serial cross-sectional analysis. https://pubmed.ncbi.nlm.nih.gov/38505738/ , last accessed 06.09.2024 10.2147/POR.S442959 PMC10949995 38505738

[CR27] MERIT-HF Study (1999) Effect of metoprolol CR/XL in chronic heart failure: metoprolol CR/XL randomized intervention trial in congestive heart failure. Lancet 353:2001–2007 10376614

[CR28] Messerli FH, Bangalore S, Bavishi C, Rimoldi SF (2018) Angiotensin-converting enzyme inhibitors in hypertension: to use or not to use? J Am Coll Cardiol 71:1474–1482 29598869 10.1016/j.jacc.2018.01.058

[CR29] Mössner J (2016) Indications, benefits and risks of proton pump inhibitors. A review after 25 years. German Med J 113:477–483 10.3238/arztebl.2016.0477 PMC4973002 27476707

[CR30] Nissen SE, Tuzcu EM, Libby P, Thompson PD, Ghali M, Garza D, Berman L, Shi H, Buebendorf E, Topol EJ, Investigators CAMELOT (2004) Effect of antihypertensive agents on cardiovascular events in patients with coronary disease and normal blood pressure: the CAMELOT study: a randomized controlled trial. JAMA 292:2217–2225 15536108 10.1001/jama.292.18.2217

[CR31] Noe LL, Vreeland MG, Pezzella SM, Trotter JP (1999) A pharmacoeconomic assessment of torsemide and furosemide in the treatment of patients with congestive heart failure. Clin Ther. 21:854–866 10397380 10.1016/s0149-2918(99)80007-1

[CR32] Olfson M, Shuai Wang, Miren Iza, Stephen Crystal, Carlos Blanco (2013) National trends in the office-based prescription of schedule ii opioids - PMC (nih.gov). J Clin Psychiatry . 74(9):932-9. 10.4088/JCP.13m08349. last accessed 04/15/2023 10.4088/JCP.13m08349 PMC8193626 24107767

[CR33] Persani L, dell’Acqua M, Ioakim S et al (2023) Factitious thyrotoxicosis and thyroid hormone misuse or abuse. Ann Endocrinol 84:367–369 10.1016/j.ando.2023.03.008 36963754

[CR34] Pilz S, Theiler-Schwarz V, Malle O et al (2020) Hypothyroidism: guidelines, new findings and clinical practice. J Clin Endocrinol Stoffw 13:88–95

[CR35] Pitt B, Poole-Wilson PA, Segal R, Martinez FA et al (2000) Effect of losartan compared with captopril on mortality in patients with symptomatic heart failure: randomised trial – the Losartan Heart Failure Survival Study ELITE II. Lancet 355:1582–1587 10821361 10.1016/s0140-6736(00)02213-3

[CR36] Rudolph UM, Enners S, Kieble M, Mahfoud F, Böhm M, Laufs U, Schulz M (2021) Impact of angiotensin receptor blocker product recalls on antihypertensive prescribing in Germany. J Human Hyperten, 35(10), 903–911. 10.1038/s41371-020-00425-z, last accessed 06.09.2024 10.1038/s41371-020-00425-z PMC8502678 33057175

[CR37] Saravanan P, Siddique H, Simmons DJ, Greenwood R, Dayan CM (2007) Twenty-four hour hormone profiles of TSH, free T3 and free T4 in hypothyroid patients on combined T3/T4 therapy. Exp Clin Endocrinol Diabetes 1 10.1055/s-2007-973071 17479444

[CR38] Scandinavian Simvastatin Survival Study Group (1994) Randomized trial of cholesterol lowering in 4444 patients with coronary heart disease. The Scandinavian Simvastatin Survival Study (4S). Lancet 344:1383–1389 7968073

[CR39] Schwabe U, Ludwig W (2020) Drug prescription report 2020: Springer, Berlin/Heidelberg

[CR40] Schwabe U, Paffrath D (1985) Drug prescription report 1985: Gustav Fischer, Stuttgart

[CR41] Schwabe U, Paffrath D (1986) Drug prescription report 1986: Gustav Fischer, Stuttgart

[CR42] Schwabe U, Paffrath D (1987) Drug prescription report 1987: Gustav Fischer, Stuttgart

[CR43] Schwabe U, Paffrath D (1988) Drug prescription report 1988: Gustav Fischer, Stuttgart

[CR44] Schwabe U, Paffrath D (1989) Arzneiverordnungsreport 1989: Gustav Fischer, Stuttgart

[CR45] Schwabe U, Paffrath D (1990) Drug prescription report 1990: Gustav Fischer, Stuttgart

[CR46] Schwabe U, Paffrath D (1991) Drug prescription report 1991: Gustav Fischer, Stuttgart

[CR47] Schwabe U, Paffrath D (1992) Arzneiverordnungsreport 1992: Gustav Fischer, Stuttgart

[CR48] Schwabe U, Paffrath D (1993) Drug prescription report 1993: Gustav Fischer, Stuttgart

[CR49] Schwabe U, Paffrath D (1994) Drug prescription report 1994: Gustav Fischer, Stuttgart

[CR50] Schwabe U, Paffrath D (1995) Drug prescription report 1995: Gustav Fischer, Stuttgart

[CR51] Schwabe U, Paffrath D (1996) Drug prescription report 1996: Gustav Fischer, Stuttgart

[CR52] Schwabe U, Paffrath D (1997) Drug prescription report 1997: Gustav Fischer, Stuttgart

[CR53] Schwabe U, Paffrath D (1998) Drug prescription report 1998: Springer, Berlin/Heidelberg

[CR54] Schwabe U, Paffrath D (1999) Drug prescription report 1999: Springer, Berlin/Heidelberg

[CR55] Schwabe U, Paffrath D (2000) Drug prescription report 2000: Springer, Berlin/Heidelberg

[CR56] Schwabe U, Paffrath D (2001) Drug prescription report 2001: Springer, Berlin/Heidelberg

[CR57] Schwabe U, Paffrath D (2002) Drug prescription report 2002: Springer, Berlin/Heidelberg

[CR58] Schwabe U, Paffrath D (2003) Drug prescription report 2003: Springer, Berlin/Heidelberg

[CR59] Schwabe U, Paffrath D (2004) Drug prescription report 2004: Springer, Berlin/Heidelberg

[CR60] Schwabe U, Paffrath D (2005) Drug prescription report 2005: Springer, Berlin/Heidelberg

[CR61] Schwabe U, Paffrath D (2006) Drug prescription report 2006: Springer, Berlin/Heidelberg

[CR62] Schwabe U, Paffrath D (2007) Drug prescription report 2007: Springer, Berlin/Heidelberg

[CR63] Schwabe U, Paffrath D (2008) Drug prescription report 2008: Springer, Berlin/Heidelberg

[CR64] Schwabe U, Paffrath D (2009) Drug prescription report 2009: Springer, Berlin/Heidelberg

[CR65] Schwabe U, Paffrath D (2010) Drug prescription report 2010: Springer, Berlin/Heidelberg

[CR66] Schwabe U, Paffrath D (2011) Drug prescription report 2011: Springer, Berlin/Heidelberg

[CR67] Schwabe U, Paffrath D (2012) Drug prescription report 2012: Springer, Berlin/Heidelberg

[CR68] Schwabe U, Paffrath D (2013) Drug prescription report 2013: Springer, Berlin/Heidelberg

[CR69] Schwabe U, Paffrath D (2014) Drug prescription report 2014: Springer, Berlin/Heidelberg

[CR70] Schwabe U, Paffrath D (2015) Drug prescription report 2015: Springer, Berlin/Heidelberg

[CR71] Schwabe U, Paffrath D (2016) Drug prescription report 2016: Springer, Berlin/Heidelberg

[CR72] Schwabe U, Paffrath D, Ludwig W, Klauber J (2017) Drug prescription report 2017: Springer, Berlin/Heidelberg

[CR73] Schwabe U, Paffrath D, Ludwig W, Klauber J (2018) Drug prescription report 2018: Springer, Berlin/Heidelberg

[CR74] Schwabe U, Paffrath D, Ludwig W, Klauber J (2019) Drug prescription report 2019: Springer, Berlin/Heidelberg

[CR75] Sleight P (2000) The HOPE study (heart outcomes prevention evaluation). J Renin-Angiotensin-Aldoster Syst 1(1):18–20. 10.3317/jraas.2000.007 10.3317/jraas.2000.002 11967789

[CR76] Statement by the Drug Commission of the German Medical Association (2006) Drug prescriptions. Recommendations for rational pharmacotherapy. 21st edition, Deutscher ÄrzteVerlag Cologne, pp 907-921

[CR77] Taylor PN, Iqbal A, Minassian C, Sayers A, Draman MS, Greenwood R, Hamilton W, Okosieme O, Panicker V, Thomas SL, Dayan C (2014) Falling threshold for treatment of borderline elevated thyrotropin levels-balancing benefits and risks: evidence from a large community-based study. JAMA Intern Med 174:32–39 24100714 10.1001/jamainternmed.2013.11312

[CR80] Topliss DJ, Soh SB (2013) Use and misuse of thyroid hormone. Singapore Med J 54:406–410 23900472 10.11622/smedj.2013143

[CR81] Vardarli I, Brandenburg T, Hegedüs L et al (2022) A questionnaire survey of German thyroidologists on the use of thyroid hormones in hypothyroid and euthyroid patients: the THESIS (treatment of hypothyroidism in europe by specialists: an international survey) collaborative. Exp Clin Endocrinol Diabetes 130:577–586 35640637 10.1055/a-1832-0644

[CR82] Wargo KA, Banta WM (2009) A comprehensive review of the loop diuretics: should furosemide be the first line? Ann Pharmacother 43:1836 19843838 10.1345/aph.1M177

[CR83] Whelton P, Carey R, Aronow W et al (2018) ACC/AHA/AAPA/ABC/ACPM/AGS/APhA/ASH/ASPC/NMA/PCNA Guideline for the prevention, detection, evaluation, and management of high blood pressure in adults: a report of the American College of Cardiology/American Heart Association task force on clinical practice guidelines. JACC. 71(19):127–248. 10.1016/j.jacc.2017.11.006 10.1016/j.jacc.2017.11.006 29146535

[CR84] Williams B, Mancia G, Spiering W, Agabiti Rosei E, Azizi M, Burnier M, Clement DL, Coca A, de Simone G, Dominiczak A, Kahan T, Mahfoud F, Redon J, Ruilope L, Zanchetti A, Kerins M, Kjeldsen SE, Kreutz R, Laurent S, Lip GYH, McManus R, Narkiewicz K, Ruschitzka F, Schmieder RE, Shlyakhto E, Tsioufis C, Aboyans V, Desormais I (2018) ESC Scientific Document Group (2018): 2018 ESC/ESH Guidelines for the management of arterial hypertension: the task force for the management of arterial hypertension of the European Society of Cardiology (ESC) and the European Society of Hypertension (ESH). European Heart Journal 39(33):3021–3104. 10.1093/eurheartj/ehy339 30165516 10.1093/eurheartj/ehy339

